# Priming effect of ascorbic acid on the growth and biomass of quinoa under saline conditions

**DOI:** 10.3389/fpls.2025.1600423

**Published:** 2025-05-30

**Authors:** Sulaiman Shah, Yaseen Khan, Zijun Cheng, Mohammed Bouskout, Tao Zhang, Hong Yan, Mingming Wang

**Affiliations:** ^1^ School of Life Sciences, Northeast Normal University, Changchun, China; ^2^ Northeast Institute of Geography and Agroecology, Chinese Academy of Sciences, Changchun, China; ^3^ Jilin Da'an Farmland Ecosystem National Field Scientific Observation and Research Station, Jilin, Da’an, China; ^4^ National Key Laboratory of Black Land Protection and Utilization, Changchun, China; ^5^ National Saline-Alkali Land Comprehensive Utilization Technology Innovation Center, Northeast Saline-Alkali Land Sub-Center, Jilin, Da’an, China; ^6^ Center for Eco-Environment Restoration Engineering of Hainan Province, School of Ecology, Hainan University, Haikou, China; ^7^ Laboratory of Water Sciences, Microbial Biotechnologies, and Natural Resources Sustainability, Unit of Microbial Biotechnologies, Agrosciences, and Environment, Labeled Research Unit-CNRST N◦4, Faculty of Sciences Semlalia, Cadi Ayyad University, Marrakesh, Morocco

**Keywords:** ascorbic acid, *Chenopodium quinoa*, photosynthetic efficiency, antioxidant response, ion homeostasis, salt stress tolerance

## Abstract

Ascorbic acid (ASA) is often recommended to mitigate the effects of saline stress on crop growth. However, no such research exists on its priming effect on the growth of quinoa (*Chenopodium quinoa* Willd.). Thus, the main goal of this study was to evaluate the potential benefits of ASA (0.1 and 0.5 μM) against salt-induced stress in quinoa seedlings. The results showed that ASA significantly improved germination, and biomass, especially fresh weight (≥47.14%) and dry weight (≥83.33%) even higher than CK, indicating enhanced plant vigor under such salt stress of 200 mM. Additionally, ASA-treated plants enhanced chlorophyll and carotenoid biosynthesis, with low ASA increased carotenoids by 95.45%, improving photosynthesis. Furthermore, ASA enhanced gas exchange, non-photochemical quenching (NPQ), and antioxidants enzyme activities, suggesting improvement in energy dissipation and potential support for oxidative stress tolerance. Notably, metabolic indicators, especially proline (≥29.89%) showed higher levels, indicating enhanced osmotic adjustment. Moreover, ASA effectively mitigated sodium (Na^+^) and chloride (Cl^−^) in roots through potassium (K^+^) uptake by at least 93.41% and elevated K^+^ levels by 99.76% in shoots, underscoring its role in mediating ion homeostasis under salinity. This study provides the first evidence that ASA could enhance biological, physiological and biochemical responses in quinoa. Applying ASA at 0.1 μM/L is feasible and effective as a priming concentration under suitable salt stress conditions.

## 1 Introduction

Soil salinization has become a global problem affecting around one third of the cultivable area worldwide (Kazemi et al., 2022). It is estimated that more than 800 million hectares of land are affected by salinity (Zhao et al., 2023). It also poses serious threats to social resources, population, the environment, and global food security (Zhang C. et al., 2019). Salinity represents a major threat to agriculture, as it severely impacts plant growth and productivity. It has significantly reduced crop growth and yields in crops such as wheat (*Triticum aestivum*), maize (*Zea mays*), rice (*Oryza sativa*), and soybean (*Glycine max*), as reported previously (Kazemi et al., 2022). Salinity disrupts plant physiological mechanisms because of the higher availabilities of sodium (Na^+^) and chloride (Cl^−^) ions, leading to osmotic stress and reducing water availability in plant tissues (Rodríguez Coca et al., 2023; Zhao et al., 2023). Imbalances in nutrient ions, such as Na^+^ and potassium (K^+^), lead to the excessive production of reactive oxygen species (ROS) (Xiong et al., 2020), damaging cellular components through lipid peroxidation and nucleic acid degradation (Ahmad and Anjum, 2023; Sadia et al., 2023). Thus, plants begin to wilt, and a reduction in growth occurs. In addition, salinity affects plant health and soil quality by disrupting the root air–water balance and degrading the soil structure (El Sebai et al., 2016). In plants, salt stress reduces antioxidant enzymatic activities such as superoxide dismutase (SOD), peroxidase (POD), and catalase (CAT), and suppresses plant immunity against biotic and abiotic stress (Ludwiczak et al., 2021). However, some studies report that salt stress could also upregulate alternative oxidase (AOX) defenses as a compensatory mechanism (Sousa et al., 2022; Spormann et al., 2024). Moreover, salinity stress adversely impacts phytohormones such as abscisic acid (ABA), gibberellins (GAs), salicylic acid (SA), and auxin production, as indicated previously (Duan et al., 2023). Currently, various strategies have been applied to reduce or completely mitigate salinity stress, including introducing new salt-tolerant genotypes, seed treatments, and biostimulant applications (Bouzroud et al., 2023). As an important biostimulator, ascorbic acid (ASA) is a water-soluble antioxidant naturally produced in plants (Hernández et al., 2023). Recently, some studies have indicated that it could alleviate various abiotic stresses, including salinity, by influencing ion homeostasis and maintaining physiological responses to salt stress (Zhang C. et al., 2019; Naz et al., 2016; El-Hawary et al., 2023). In addition, some investigations demonstrated that it could positively affect plant growth through variations in metabolic pathways, such as the Calvin cycle, tricarboxylic acid cycle, proline biosynthesis pathway, glycine betaine biosynthesis pathway, ascorbate-glutathione cycle, and salt overly sensitive pathway (Sadak et al., 2023; Hasanuzzaman et al., 2023). These pathways are closely associated with photosynthesis, antioxidant defense mechanisms, and ion homeostasis, and their enhancement enables plant to better tolerate salinity stress, while maintaining normal growth as indicated by Akram et al. (2017). In addition, several studies have reported that ASA is an efficient and cost-effective approach for mitigating salt stress in crops such as wheat, maize, and rice (Zhang G. et al., 2019). However, its role in quinoa remains unexplored, particularly during the seedling stage, a critical phase for establishing crop resilience under stress. Understanding the effects of ASA at this early growth stage is essential, as seedling vigor strongly influences overall plant development, salt tolerance, and yield potential under saline conditions.

Quinoa (*Chenopodium quinoa* Willd.) is a pseudo-cereal crop belonging to the Amaranthaceae family. It is highly nutritious and usually grown for protein-rich, gluten-free seeds (Keutgen and Pawelzik, 2009). It can be used in salads, porridges, and flour-based products for human nutrition. The leaves are used as a animals feed and for medicinal purposes such as diabetes control, by pharmacologists. However, cultivation, genetic diversity, and biochemical composition depend on the area and quinoa variety. Although quinoa is recognized for its tolerance to environmental stresses. However, salinity still significantly affects its growth and chemical composition (Song et al., 2019). The seedling stage is particularly sensitive to salt stress, and early damage can impair plant development and productivity. To address this, the present study aimed to investigate the effects of exogenous ASA application on quinoa seedlings under such conditions. The objective of the study was to evaluate how ASA modulates key physiological, biochemical and growth-related parameters under salt stress. Specifically, we examined changes in germination, biomass accumulation, photosynthetic traits, ion balance, antioxidant activity, and osmotic adjustment to better understand the mechanisms through which ASA supports stress resilience at this critical stage. Investigating the role of ASA in early growth could enhance crop resilience and improve agriculture sustainability under saline conditions.

## 2 Materials and methods

### 2.1 Plant material

The quinoa seeds used in this study were provided by the Gansu Academy of Agricultural Sciences, China. Before sowing, the seeds were washed with 10% sodium hypochlorite (NaClO) for 10 minutes to remove seed-borne pathogens. The sand was sieved through a 2-mm mesh sieve and washed twice with distilled water to remove dust and impurities. The pots with a 13 cm lower diameter, 16 cm upper diameter, and 16 cm height were selected for growing quinoa plants. The drainage holes of the pots were covered with a small plastic mesh to retain the sand and ensure proper drainage before adding a total of 2.5 kg of sand into each pot. In each pot, ten healthy and uniform quinoa seeds were sown at a depth of 2 cm and 3 cm apart. After sowing, the seeds were gently covered with a thin layer of sand to protect them from environmental factors.

### 2.2 Experimental design and salt stress treatments

The experiment was conducted in a greenhouse at the School of Life Sciences, Northeast Normal University, Changchun, China, from April 24 to May 25, 2023. The greenhouse maintained an average temperature of 25°C, with a photoperiod of 14 hours of light and 10 hours of darkness. A total of six treatments were applied to thirty-six pots, with six replicates per treatment (n=36). The seedlings were irrigated twice daily, and each pot received 200 ml of water throughout the experiment. During the first week after germination, seedlings were supplied with a half-strength Hoagland solution (HHS), followed by a gradual increase to full strength until harvest. The seedling stage is critical for plant growth, as it determines the foundation for later development and productivity as reported by Zarbakhsh and Shahsavar (2023). After reaching the four-leaf stage, only three healthy plants were retained per pot to ensure consistency. At the tillering stage (1 month after sowing), the seedlings were exposed to salt stress using a high concentration of 200 mM NaCl. The salt concentration was gradually raised by 50 mM per day until the final concentration was reached. In parallel, ASA at low (0.1 μM) and high (0.5 μM) concentrations was applied via soil drench to seedlings under both salt stressed and non-stressed conditions. The plants were harvested after 7 days of exposure to the final salt concentration. FW weight was immediately recorded. To determine dry weight (DW) and relative water content (RWC), samples were oven-dried at 60°C for two days. The dried samples were then ground into a fine powder and stored for ion analysis, while fresh tissue was used for enzymatic and physiological assays. The treatments included T_1_ (control; CK), T_2_ (0.1 μM ASA), T_3_ (0.5 μM ASA), salt stress T_4_ (200 mM NaCl), T_5_ (0.1 μM ASA + 200 mM), T_6_ (0.5 μM ASA + 200 mM). In this study, 200 mM NaCl concentration was selected due to the following reasons: (1) this level of salinity stimulate severe salinity stress, which is well-documented in the literature to significantly inhibit seed germination and early seedling growth of quinoa cultivars (Keutgen and Pawelzik, 2009; Shah et al., 2020); (2) it ensures alignment with protocols and enables effective comparison with previous studies on salt tolerance mechanisms of quinoa; (3) it helps identify the threshold at which the adaptative mechanisms as such ion regulation, and osmotic adjustment activated in quinoa; and (4) it replicate real-world saline conditions, similar to those in arid and semi-arid regions, where NaCl levels often exceeds 100 mM (Chauhan, 2022).

### 2.3 Seed germination parameters

Seed germination was analyzed using a factorial experimental design. The seeds were disinfected in 10% sodium hypochlorite for 10 minutes, followed by thorough rinsing with sterile distilled water twice. Fifty uniform-sized quinoa seeds were placed in 9 cm diameter Petri dishes containing two layers of filter paper. The filter papers were moistened with 5 ml of treatment solutions, including distilled water (control), salt solution (200 mM NaCl), ASA solutions (0.1 and 0.5 μM) and combined ASA and salt solutions, following the same treatment arrangement as described above for quinoa seedling growth. A total of 24 Petri dishes (three replicates per treatment) were placed in a growth chamber maintained at 25°C under a 16-hour light/8-hour dark cycle for five days. Germination was recorded daily, with seeds considered germinated when the radicle reached a length of 2 mm. Additionally, at 6-hour intervals during germination, shoot length (SL) and root length (RL) of ten randomly selected seedlings per Petri dish were measured. The following germination parameters were evaluated:

#### 2.3.1 Germination percentage and germination energy

Germination percentage (GP) and germination energy (GE) were calculated according to the method of Dehnavi et al. (2020) using the following formulae:


$$\text{GP}=\left(\frac{\text{Number of germinated seeds}}{\text{Total number of seeds tested}}\right)\times 100$$

$$\text{GE}=\left(\frac{\text{Number of seeds germinated on day X}}{\text{Total number of seeds tested}}\right)\times 100$$

#### 2.3.2 Seed vigor index and speed of germination

The seed vigor index (SVI) and speed of germination (SP) were calculated based on the method of Kandil et al. (2012) using the following formulae:


SVI=(Average shoot length+Average root length)×Germination percentage

$$\text{SP}=\left(\frac{\text{Number of seeds germinated in }24\text{ h }}{\text{Number of seeds germinated in }72\text{ h}}\right)\text{x}100$$

#### 2.3.3 Mean germination time and germination rate index

The mean germination time (MGT) and germination rate index (GRI) were calculated based on the method of Safdar et al. (2014) using the following formulae:


$$\text{MGT }=\frac{\text{Σ}\left(\text{D}\times \text{n}\right)}{\text{Σn}}$$

where n is the number of germinated seeds on a given day, and D is the number of days since the start of germination.


$$\text{GRI }=\frac{N_{1}}{D_{1}}+\ldots +\frac{N_{L}}{D_{L}}$$

where N_1_ is the number of seeds germinated on the first day; D_1_ is the days to the first count; N_L_ is the number of seeds germinated on the last day; D_L_ ​is the days to the last count

### 2.4 Measurement of growth parameters

#### 2.4.1 Relative growth rate

Eight quinoa plants were harvested before the treatment, and their FW and DW were recorded. After 7 days of stress treatment, all plant material in each pot was collected, and the same weight measurement was performed as described above at the end of the experiment. The relative growth rate (RGR) was calculated using the following formula:


$$\text{RGR}=\frac{\text{ln }W2\;–\text{ ln }W1}{t2\;–\;t1}$$

where ln= natural logarithm; *W1*= plant weight before treatment; *W2*= plant weight after treatment; *t1*−*t2*= the duration of the treatment period (start and end times).

#### 2.4.2 Relative water content

The leaf sample was immediately weighed to determine the FW, and then placed into distilled water for 4 h at room temperature to achieve full turgidity. The turgid weight (TW) was determined immediately after blotting, and the dry weight (DW) was measured after the samples had dried for 48 h at 60°C in an oven. The RWC was calculated using the following formula:


$$\text{RWC}=\frac{\text{FW }–\text{ DW}}{\text{TW }–\text{ DW}}$$

where FW= fresh weight of the sample, TW= turgid weight of the sample, and DW= dry weight of the sample.

### 2.5 Photosynthetic parameters

#### 2.5.1 Gas exchange and chlorophyll *a* fluorescence

The photosynthetic rate (*P_N_*), stomatal conductance (*gs*), transpiration rate (*E*), water use efficiency (*WUE*), and intercellular carbon dioxide concentration (*Ci*) were measured using LI-6800 portable photosynthesis system (Li-Cor Inc., Lincoln, NE, USA) following Sánchez-Reinoso et al. (2019). The measurements were taken on mature and sun-exposed leaves during the morning hours (8:30-11:00) under clear weather conditions. The chlorophyll *a* fluorescence parameters were recorded using the LI-6800 system on the same leaves for gas exchange after 30 minutes of dark adaptation. We recorded the key parameters such as initial fluorescence (F0), maximum fluorescence (Fm), maximum quantum efficiency of PSII (Fv/Fm), potential activity (Fv/Fo), photochemical quenching (qP), non-photochemical quenching, and effective electron transport rate (ETR) following the method outlined by Sánchez-Reinoso et al. (2019).

#### 2.5.2 Photosynthetic pigments

Undamaged fresh leaves were selected for the extraction of photosynthetic pigments. A 0.1 g fresh leaf sample was weighed and cut into uniform pieces and placed in a stoppered test tube. A 10 ml mixture of 80% acetone and absolute ethanol (1:1) were added and covered with black paper and kept in the dark for 20 hours. The absorbance was measured at wavelengths of 663 nm, 645 nm, and 440 nm using a spectrophotometer (UV-754, Shanghai Precision Science Instrument Co. Ltd., Shanghai, China). The concentrations of photosynthetic pigments were determined using the following formulae:


$$Ca=9.784\;A_{663}\;–\;0.990\;A_{645}$$

$$Cb=21.426\;A_{645}\;–\;4.65\;A_{663}$$

$$Ct=Ca\;+\;Cb=5.134\;A_{663}+20.436\;A_{645}$$

$$Cc=4.695\;A_{440}\;–\;0.268\left(Ca+Cb\right)$$

where *Ca=* chlorophyll a concentration; *Cb=* chlorophyll b concentration; *Ct=* total chlorophyll concentration; *Cc=* carotenoid concentration.

### 2.6 Determination of biochemical parameters

#### 2.6.1 Soluble sugar

The soluble sugar content was determined by using anthrone colorimetry described by Chen et al. (2023). Ten mg of dried leaf sample was extracted with 10 ml of distilled water and incubated in a water bath at 70-80°C for 1 hour. The mixture was then filtered to obtain a clear extract and 5 ml of anthrone reagent was added. The mixture was heated in a water bath for 10 minutes and then cooled to room temperature. The absorbance value of the final extract was measured at a wavelength of 625 nm using a spectrophotometer (UV-754). The soluble sugar content were calculated using the following formula:


$$\text{Soluble sugar content }\left(\text{mg}/\text{g}\right)=\frac{\left(\text{C }\times \text{V}\right)}{\left(\text{W }\times \;10^{6}\right)}$$

where *C=* concentration of soluble sugar from standard curve (mg/mL), *V=* total volume of the extract (mL), and *W=* weight of dried tissue (g).

#### 2.6.2 Proline content

The proline content was quantified using the acid ninhydrin method described by Bates et al. (1973). A total of100 mg of dried leaf sample were homogenized in 5ml of 3% sulfosalicylic acid, and incubated for 2–4 hours. The mixture was then heated in a boiling water bath for 10 minutes to extract proline. The extracted solution (1 ml) was treated with 1 ml distilled water, 1 ml glacial acetic acid, and 2 ml of 2.5% ninhydrin solution. The reaction mixture was heated in boiling water bath for 60 minutes. Next, 4 ml of toluene was added to the solution after cooling and shaken to extract the red-colored proline-ninhydrin complex. The absorbance value of the supernatant was measured at wavelength of 520 nm using a UV-754 spectrophotometer. The proline content was calculated using the formula below:


$$\text{proline content }\left(\text{mg}/\text{g}\right)=\frac{\left(\text{C }\times \text{VT}\right)}{\left(\text{W }\times \;1000\right)}$$

where *C*= concentration of proline from standard curve (mg/mL), *VT=* total volume of the extract (mL), and *W=* sample dry weight (g).

#### 2.6.3 Ion contents

The dried leaf and root samples (50 mg each) were ground and extracted with 10 ml of distilled water. The samples were heated in boiling water bath for 1 hour and then allowed to stand at room temperature overnight to ensure complete extraction. The supernatant was collected by centrifugation and used for further analysis. The cation contents (Na^+^, K^+^, Ca^2+^and Mg^2+^) were measured using an atomic absorption spectrophotometer (Supper 990F, Beijing, China). The cation ratios (Na^+^/K^+^, Na^+^/Ca^2+^) were calculated to evaluate ionic balance. The anion contents (Cl^-^ and SO_4_^2-^) were determined using ion chromatography (DX-300 system, Dionex, Sunnyvale, USA) equipped with an AS4A-SC anion column, and a CDM-II conductivity detector. The mobile phase used was a mixture of Na_2_CO_3_ (1.7 mM) and NaHCO_3_ (1.8 mM), following the procedure described by Wang et al. (2015).

### 2.7 Antioxidant activities

Fresh quinoa leaf samples were rapidly frozen in liquid nitrogen, wrapped in aluminum foil, and stored at -80°C for enzymatic analysis. For enzyme extraction, 200 mg of fresh leaf tissue was homogenized in an ice-cold mortar and added to 50 mM Tris-HCl buffer (pH 7.5) containing 1 mM EDTA and 1%(w/v) polyvinylpyrrolidone (PVP) to prevent phenolic oxidation. The homogenate was centrifuged at 10,000× g for 15 minutes at 4°C, and the supernatant was collected as the enzyme extract. The activities of superoxide dismutase (SOD) and peroxidase (POD) were measured using the nitroazoblue tetrazolium (NBT) and guaiacol oxidation methods, respectively. Catalase (CAT) activity was determined by measuring the H2O2 decomposition rate as described by Luna et al. (2005). The total soluble protein of the leaf was measured using the Coomassie Brilliant Blue (CBB) method with bovine serum albumin (BSA) as the standard. The activity of various enzymes was calculated using the following formula:


$$\text{SOD activity }\left(\text{U}.{\text{ g}}^{-1\;}\text{FW}\right)=\frac{\left(\text{IA control }–\text{IA Sample}\right)}{\left(100\%\times V_{t}50\%\times 0.1\right)}\times W$$

$$\text{POD activity }\left(\text{U}.{\text{ g}}^{-1\;}\text{FW}\right)=\frac{\Delta A_{470}\times V_{t}}{W\times V_{s}\times 0.01}\times t$$

$$\text{CAT activity }\left(\text{U}.{\text{ g}}^{-1\;}\text{FW}\right)=\frac{\Delta A_{240}\times V_{t}}{W\times V_{s}\times 0.01}\times t$$

where *W*= FW of the tissue sample (g), *t*= reaction time (min), *V_t_* = total volume of enzyme extract (mL), *V_s_* = volume of enzyme extract used in the assay (mL) and *ΔA*= change in absorbance per minute at respective wavelengths.

### 2.8 Malondialdehyde content

The malondialdehyde (MDA) content in leaves was measured using the thiobarbituric acid (TBA) method described by Yasar et al. (2008). Fresh leaf samples (0.2 g) were homogenized in 1.6 ml of 10% trichloroacetic acid (TCA) and small amount of quartz sand using pre-chilled mortar and pestle on ice. The homogenate was centrifuged at 4,000× g for 10 min at 4°C. The obtained supernatant was used for measuring MDA contents. For the MDA reaction, 1.5 ml of the supernatant was mixed with 1.5 ml of 0.6% TBA solution. The mixture was incubated in a boiling water bath for 30 minutes, then quickly cooled on ice to stop the reaction. Next, the samples were centrifuged at 3,000× g for 15 minutes. The absorbance values of the supernatant were measured using a spectrophotometer (UV-754) at 532 nm, 600 nm, and 450 nm. The MDA content was calculated using the following formulae:


$$C_{\text{MDA}}=11.71\;A_{450\;nm}$$

$$C_{\text{MDA}}\;\left(\mu {\text{mol L}}^{-1}\right)=6.45\left(A_{532\;nm}–\;A_{600\;nm}\right)-0.56A_{450\;nm}$$

$$\text{MDA content }\left(\mu \text{mol}/\text{g FW}\right)=\frac{C_{\text{MDA}}\times V}{W}$$

where *V*= the total extract volume of the extract (mL), *W=* the FW of the sample (g), and *C*_MDA_= the MDA concentration.

### 2.9 Statistical analysis

Statistical analyses were performed using one-way ANOVA for single-factor treatments and two-way ANOVA for evaluating interactions between salt stress and melatonin levels, using SPSS version 25.0. Tukey’s test was used for multiple comparisons to determine significant differences among treatments, with the significance level set at *P<* 0.05. The discrepancies between treatments were identified by using six biological replicates for each treatment. Important variables were identified, and their scores were obtained by the principal component analysis (PCA). GraphPad Prism (version 8.02) was used for data processing and figure creation.

## 3 Results

### 3.1 The effects of ASA on seed germination of quinoa under salt stress

**Figure 1:**
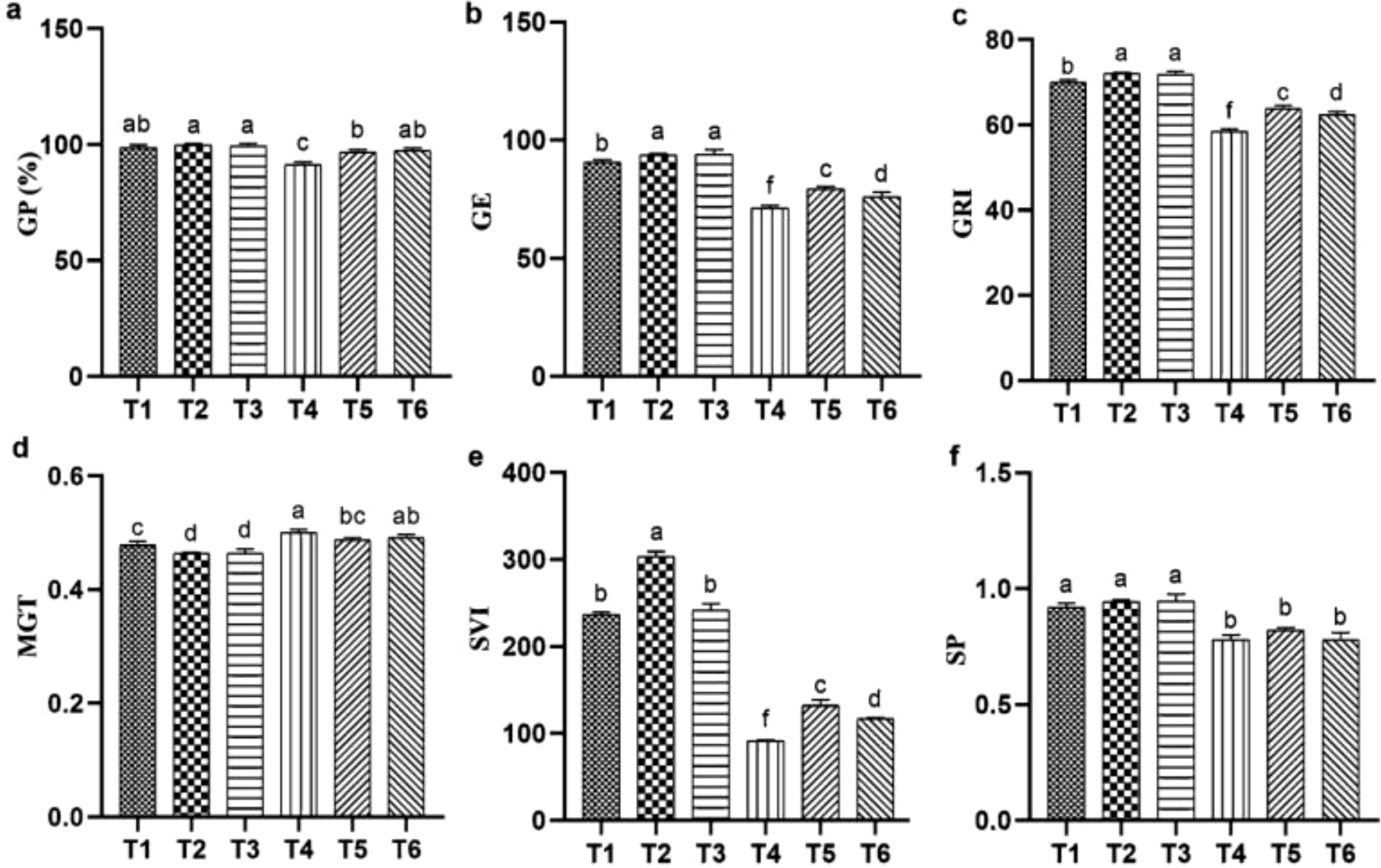
Influence of ASA and salt stress on GP **(a)**, GE **(b)**, GRI **(c)**, MGT **(d)**, SVI **(e)**, and SP **(f)** in quinoa seeds under salinity stress. Bars represent the mean ± S.E. (N=3); significant differences (*P<* 0.05) are marked by distinct letters (a–e).

In this study, T_4_ (salt stress) negatively affected the germination of quinoa compared with the T_1_ (CK). However, the application of T_2_, T_3_ (ASA treatments alone) significantly enhanced germination parameters relative to the CK. Similarly, T_5_, and T_6_ (ASA treatments combined with salt stress) showed a positive effect. Specifically, parameters such as GP, GE, GRI, and SVI were significantly improved by 24.89%, 4.44%, 5.45%, and 3.57%, respectively, compared with salt stress alone, as shown in **Figures 1a–c, e**. However, no significant effect of ASA application on MGT was observed (**Figure 1d, f**). In addition, ASA promoted radicle and plumule growth over the five-day observation period, as this growth was markedly reduced by salinity stress.

### 3.2 The effect of ASA on growth and biomass of quinoa under salt stress

**Figure 2:**
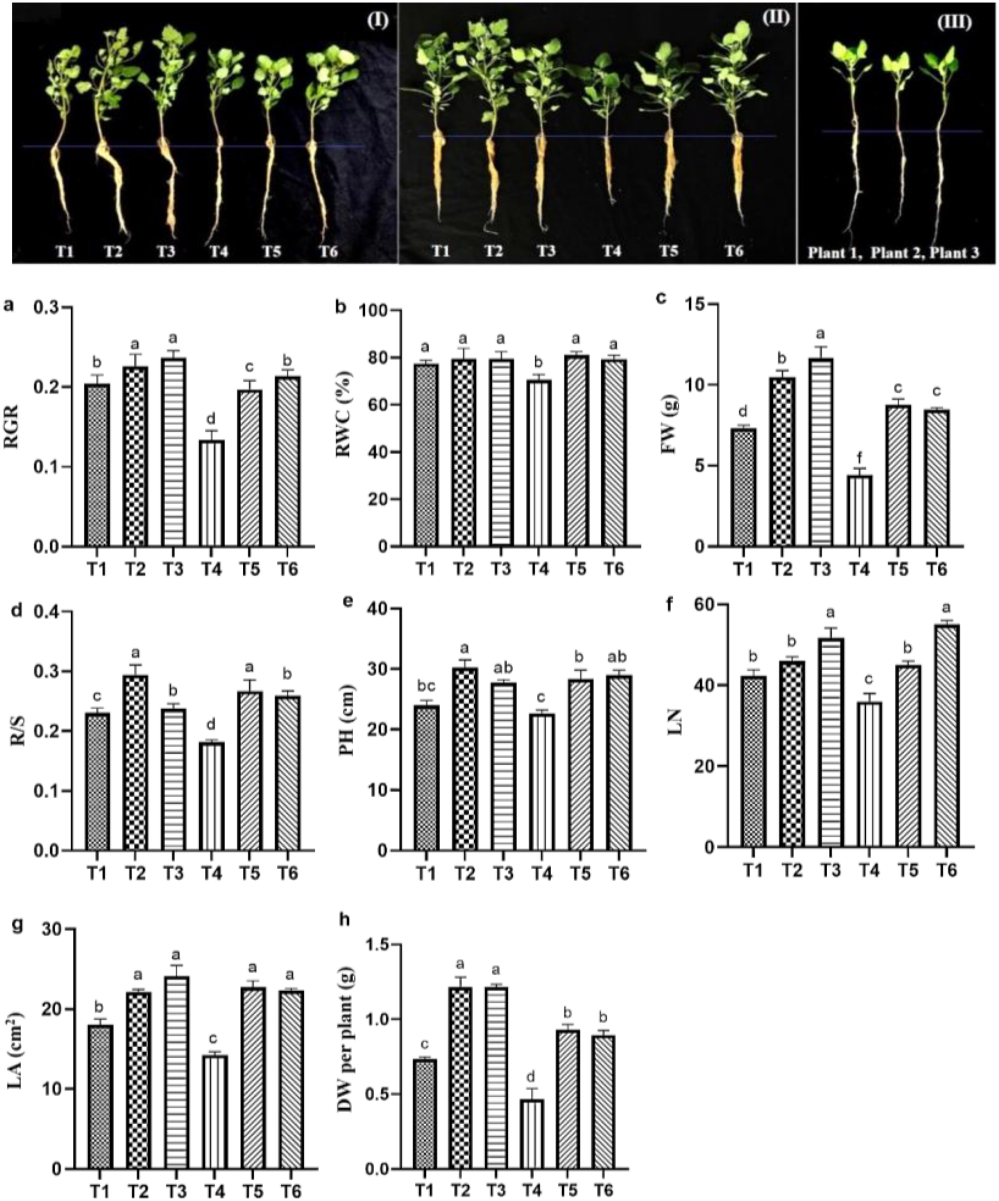
Effect ASA application on quinoa seedling growth under salt stress **(I, II)** and growth of quinoa seedlings before exposure to stress **(III)**. The growth parameters include RGR **(a)**, RWC **(b)**, FW per plant **(c)**, R/S **(d)**, PH **(e)**, LN **(f)**, LA **(g)**, and DW per plant **(h)**. Bars represent the mean ± S.E. (n=6); significant differences (*p<* 0.05) are shown by distinct letters (a–d) in the figure.

The effects of ASA, salt stress and their interaction on quinoa seedling biomass under salt stress are provided in **Table 1**. Salt stress significantly reduced quinoa biomass and growth parameters compared with the CK treatment. Specifically, RWC was significantly decreased by salt stress alone as compared to other treatments as shown in **Figure 2**. However, both ASA treatments, with and without salt stress, positively affected these parameters. The DW increased by 85.41% and 83.33% under ASA treatments alone compared with the CK treatment (**Figure 2h**). Similarly, ASA treatments enhanced parameters such as relative growth rate (RGR), leaf number (LN), and plant height (PH), and reduced the root-to-shoot ratio (R/S) under salt stress compared with salt treatment alone (**Figures 2a–e**). Moreover, the FW increased by 49.54% and 47.14%, and the LA of quinoa by 53.15% and 53.06% under both ASA concentrations combined with salt stress, respectively, compared with salt treatment alone.

**Table 1:** Two-way analysis of gas exchange and growth parameters of quinoa.

Treatments	*P_N_*	*E*	*gs*	*Ci*	*WUE*	RGR	RWC	FW	R/S
ASA	***	***	**	*	***	***	***	***	***
NaCl	**	**	*	***	ns	*	*	**	*
ASA×NaCl	***	***	***	**	**	**	**	**	***

Significant differences are marked with asterisks: ∗*P<* 0.05, ∗∗*P<* 0.01, and ∗∗∗*P<* 0.001. Differences with *P >* 0.05 are regarded as non-significant.

### 3.3 The effect of ASA on photosynthetic characteristics of quinoa under salt stress

#### 3.3.1 Changes in gas exchange parameters

**Figure 3:**
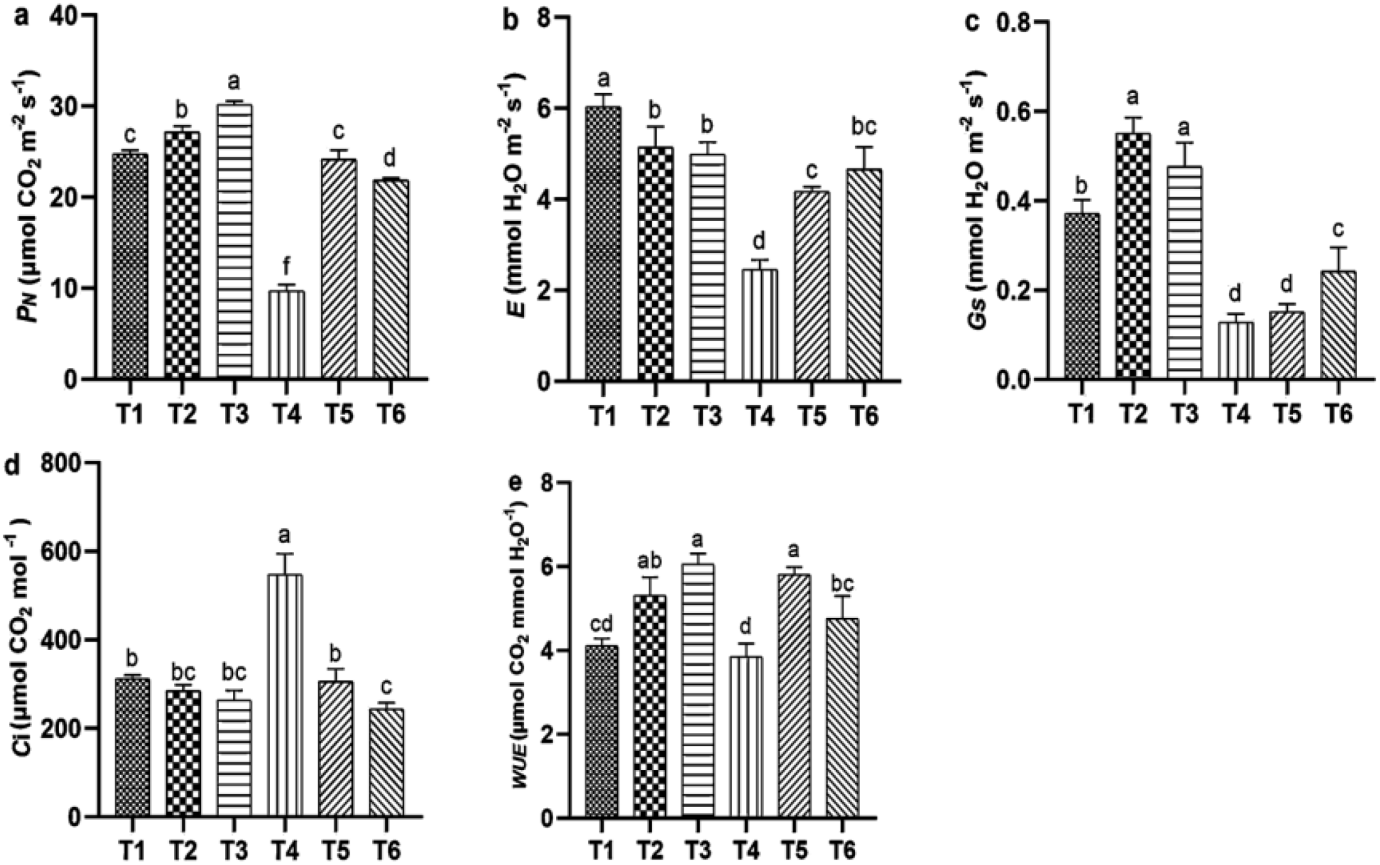
Influence of ASA and salt stress on *P_N_* **(a)**, *E* **(b)**, **(c)**, *Ci* **(d)**, and *WUE* **(e)** in quinoa seedlings. Bars represent the mean ± S.E. of six replicates; significant differences (*p<* 0.05) are marked by letters (a–f).

Salt stress significantly reduced gas exchange parameters, while ASA application under salt stress improved these parameters. Salt stress alone significantly reduced *E* (55.34%) and *C_i_* (76.66%) compared with the CK treatment. However, positive changes in these parameters were observed with the ASA treatment alone or in combination with salt stress. The ASA treatments alone significantly influenced *E*, *P_N_*, g_s_, and *WUE*, compared with CK, as shown in **Figures 3a–c**. The high-ASA treatment significantly reduced *Ci* by 53.83% when combined with salt stress, compared with salt stress alone (**Figure 3d**). Moreover, the high-ASA treatment alone significantly enhanced *WUE* by 46.34% compared with CK, whereas the low-ASA treatment combined with salt stress increased *WUE* by 52.45% compared with salt stress alone (**Figure 3e**).

#### 3.3.2 Change in chlorophyll *a* fluorescence

**Figure 4:**
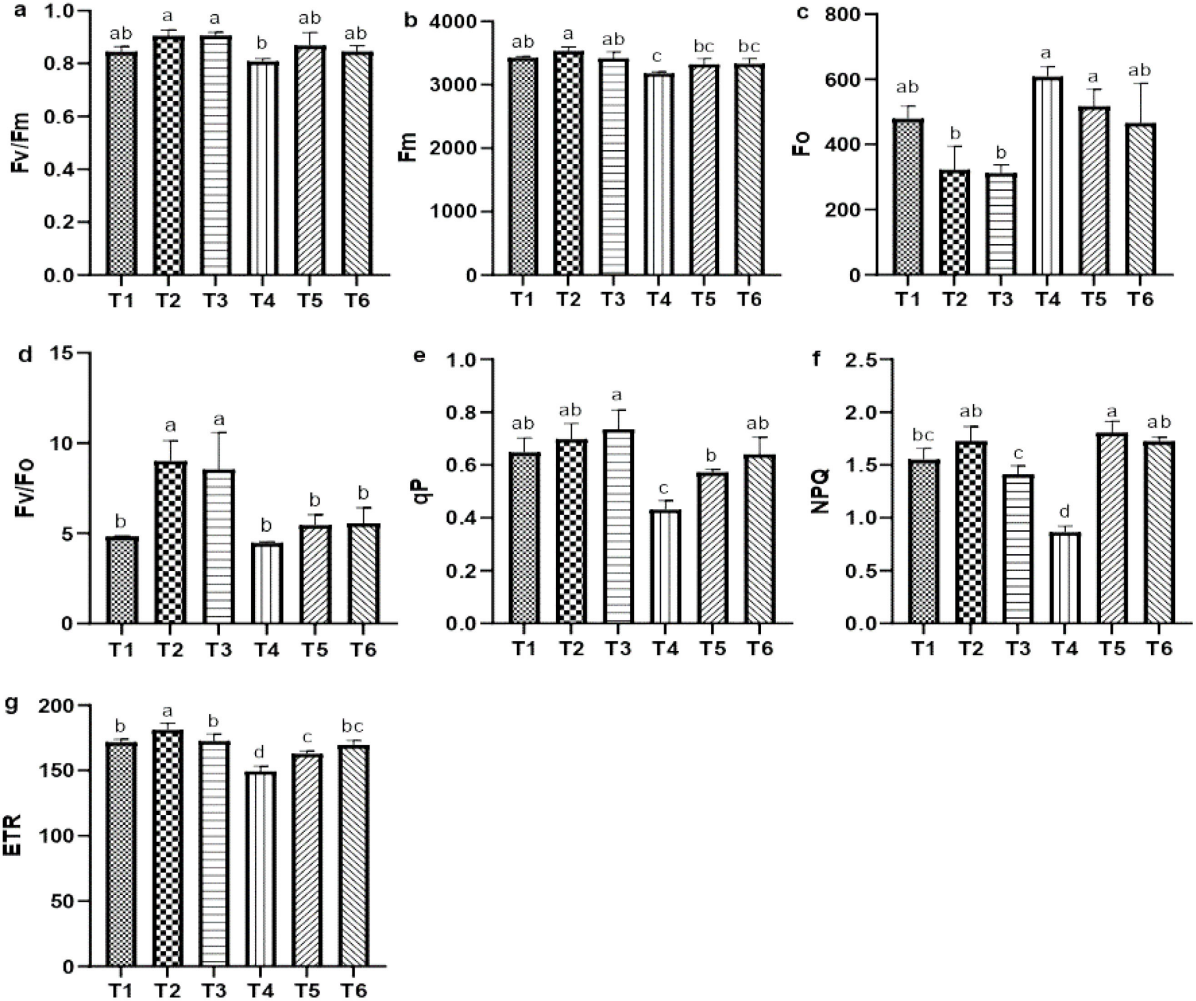
Influence of AsA and salt stress on the Fv/Fm **(a)**, Fm **(b)**, Fo **(c)**, Fv/Fo **(d)**, qP **(e)**, NPQ **(f)**, and ETR **(g)** in quinoa. Bars represent the mean ± S.E. (n=6); letters (a–c) show significance (*P<* 0.05).

Salt stress significantly impaired chlorophyll *a* fluorescence parameter, including the maximum quantum efficiency of PSII (Fv/Fm), maximum fluorescence (Fm), potential activity, photochemical quenching (qP), non-photochemical quenching, and effective electron transfer rate (ETR), compared with CK. Notably, NPQ exhibited a substantial decreased of 79.66% relative to CK; however, ASA treatments positively influenced these parameters in quinoa (**Figures 4a–c**). Both ASA treatments alone enhanced potential activity (Fv/Fo) by 61.34% and 50.11%, respectively, compared with CK (**Figure 4d**). Conversely, ASA combined with salt stress significantly increased qP by 38.81% and 52.48%, NPQ by 95.18% and 92.25%, and ETR by 12.44% and 16.03%, respectively, compared with salt stress alone (**Figures 4e–g**).

#### 3.3.3 Changes in photosynthetic pigments

**Figure 5:**
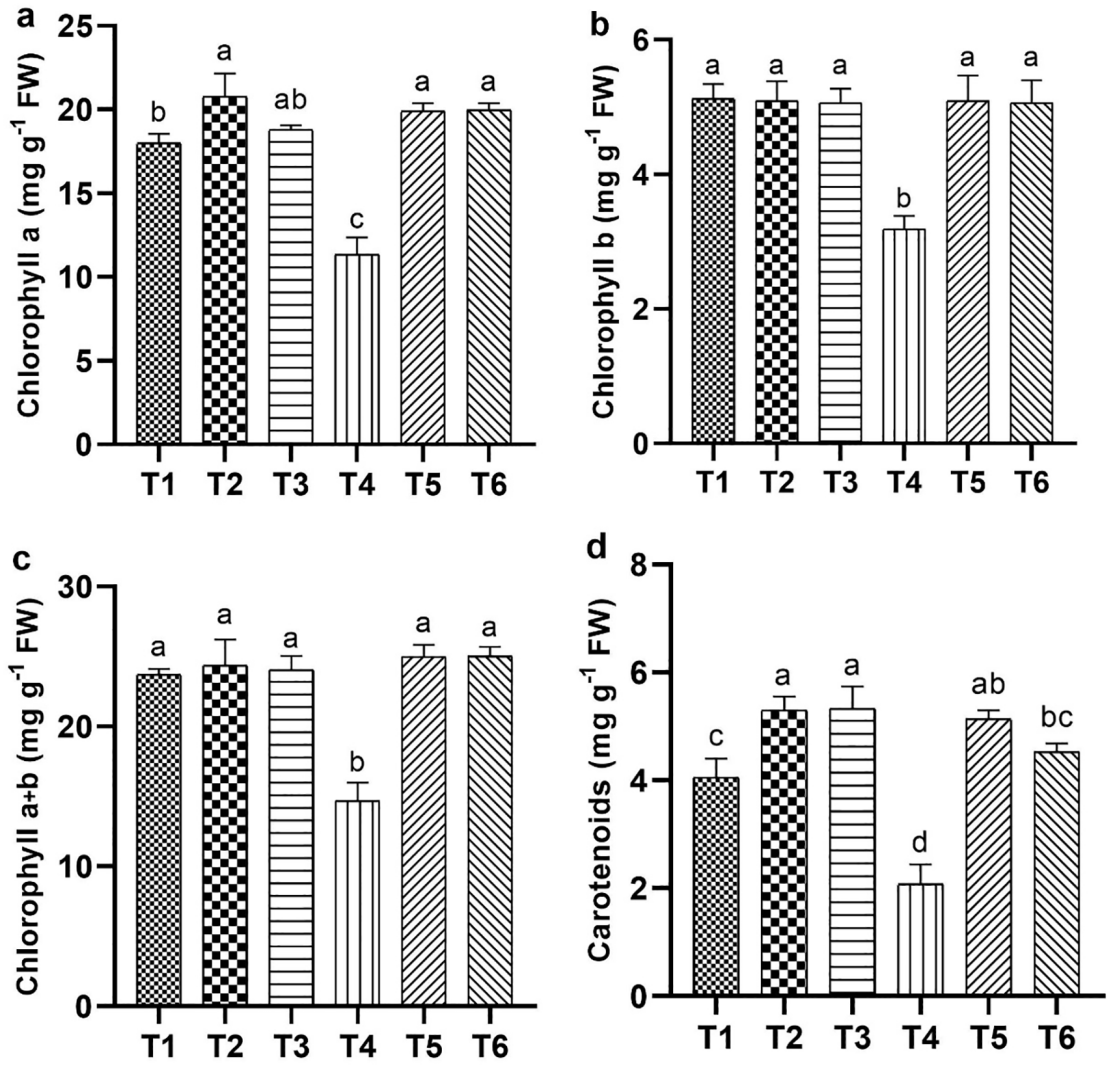
Influence of ASA and salt stress on photosynthetic pigments in quinoa seedlings: chlorophyll a **(a)**, chlorophyll b **(b)**, total chlorophyll **(c)**, and carotenoids **(d)**. Bars represent the mean ± S.E. (n=6); significant variations (*P<* 0.05) are shown by distinct letters (a–d).

Salt stress significantly reduced the chlorophyll and carotenoid levels, specifically the chlorophyll b and total chlorophyll contents, which decreased by 40.13% and 37.53%, respectively, compared with CK. In contrast, the ASA treatments, with and without salt stress, positively influenced these contents. High-ASA treatment under salt stress increased chlorophyll a content by 83.63% compared with salt stress alone (**Figure 5a**). Similarly, ASA treatments under salt stress significantly elevated the levels of chlorophyll b by 66.56% and 65.66%, total chlorophyll by 78.58% and 77.97%, and carotenoids by 95.45% and 86.36%, respectively, relative to salt stress alone (**Figures 5b–d**).

### 3.4 The effect of ASA on the biochemical indexes of quinoa under salt stress

#### 3.4.1 Change in soluble sugar and proline contents

**Figure 6:**
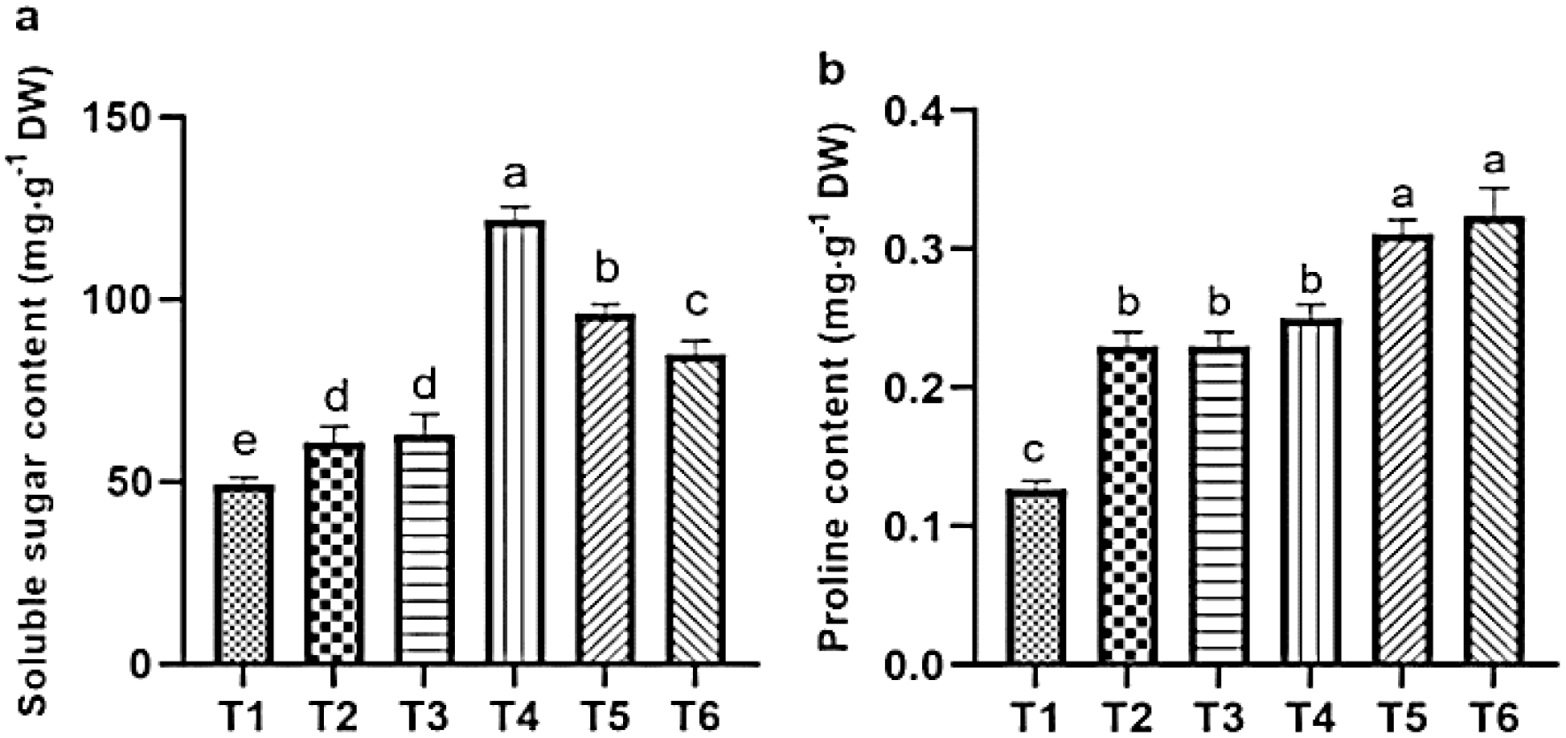
Influence of ASA and salt stress on the soluble sugar **(a)** and proline **(b)** contents in quinoa seedlings. Bars represent the mean ± S.E. (n=6); letters (a–d) show significant differences (*P<* 0.05).

Salt stress significantly elevated the levels of soluble sugars and proline, which increased by 62.55% and 48.78%, respectively, compared with the CK. Although ASA treatments alone also increased soluble sugar contents by 28.83% and 22.66%, and proline by 65.77% and 63.17%, respectively relative to CK. The increased in sugars was lower than that observed under salt stress alone. However, under salt stress conditions, the application of ASA further improved these osmolytes. Specifically, the low-ASA treatment increased soluble sugars by 22.50%, while both low and high ASA treatments boosted the proline levels by 29.89% and 31.39%, respectively, compared to salt stress alone (**Figures 6a, b**).

### 3.5 The effect of ASA on the ion contents in quinoa seedlings under salt stress

#### 3.5.1 Change in ion contents in leaves

**Figure 7:**
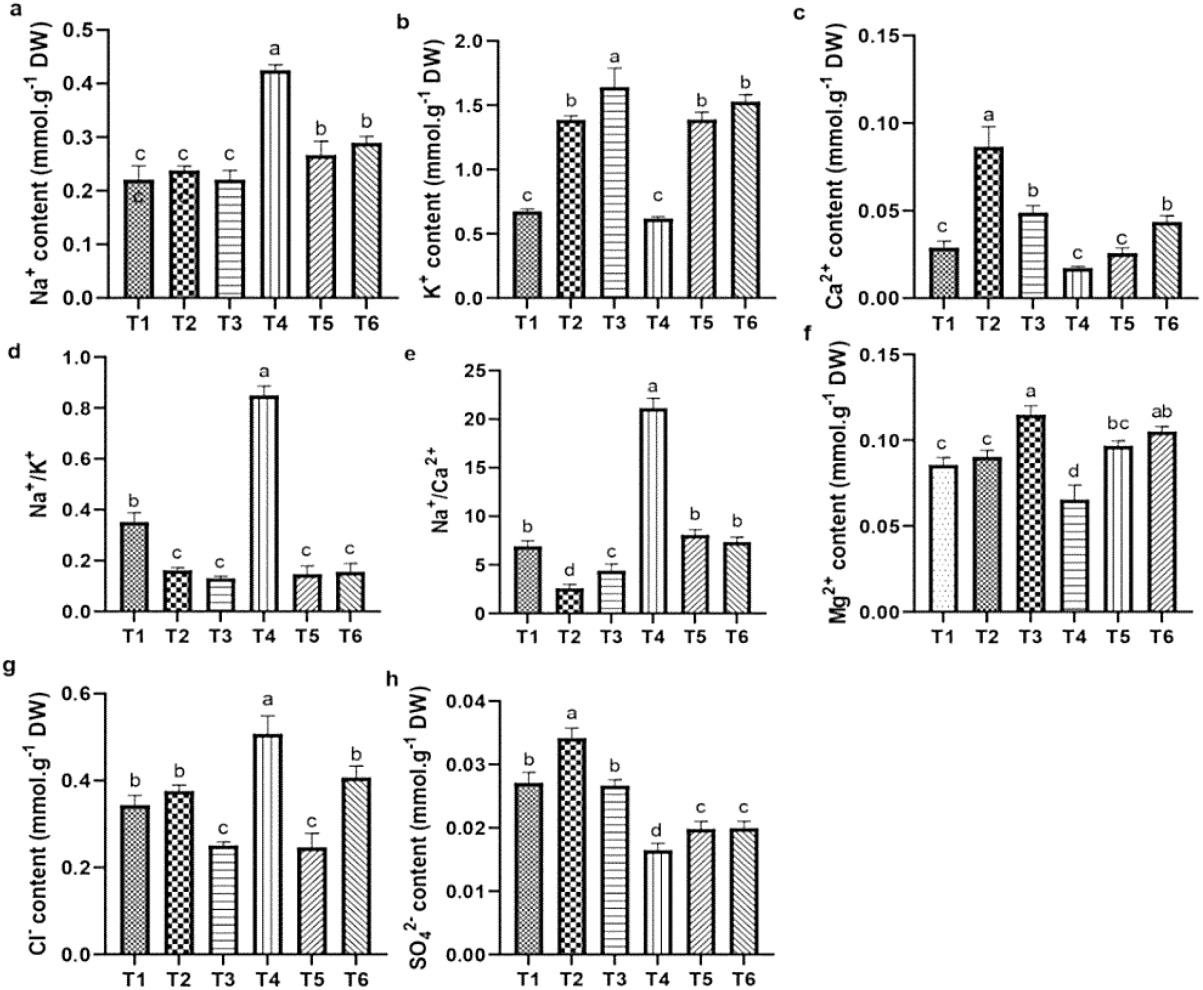
Influence of AsA and salt stress on Na^+^ **(a)**, K^+^ **(b)**, Ca^2+^ **(c)**, Na^+^/K^+^ **(d)**, Na^+^/Ca^2+^ **(e)**, Mg^2+^ **(f)**, Cl^-^ **(g)**, and SO_4_^2-^ **(h)** in quinoa leaves. Bars represent the mean ± S.E. (n=6), with significant variations (*P<* 0.05) represented by distinct letters (a–d).

Salt stress caused significant reductions in the levels of Ca²^+^, K^+^, Mg²^+^, and SO_4_²^−^ while it increased the levels of Na^+^ and Cl^−^ and Na^+^/Ca²^+^, and Na^+^/K^+^ ratios in quinoa leaves, compared to CK. However, ASA treatments alleviated these effects. Notably, the ASA treatments alone significantly increased the Ca²^+^, K^+^, SO_4_²^−^, and Mg²^+^ levels while reducing the Na^+^/K^+^ and Na^+^/Ca²^+^ ratios compared with the CK treatment (**Figures 7d, e**). Under salt stress, the ASA treatments significantly improved K^+^ by 93.41% and 99.76%, Mg²^+^ by 42.56% and 65.12%, SO_4_²^−^ by 28.48% and 34.78%, and Ca²^+^ by 94.28% and 49.04%, while they decreased Na^+^ by 41.14% and 33.79%, and Cl^^−^^ by 48.78% and 21.45%, respectively, compared with salt treatment alone (**Figures 7a–c, f**). The interaction between ASA and salt stress affected the Na^+^, K^+,^ Ca²^+^, Mg²^+^, and Cl^−^ levels in quinoa, although NaCl alone did not significantly impact K^+^ levels (**Table 2**).

**Table 2:** Two-way analysis of ion contents in the root and leaves of quinoa seedlings.

Plant part	Treatments	Na^+^	K^+^	Ca^2+^	Mg^2+^	Cl^-^	SO_4_^2-^
Leaf	ASA	*	***	***	***	**	***
	NaCl	***	ns	*	**	***	*
	ASA×NaCl	*	**	**	*	**	*
Root	ASA	ns	***	***	***	*	***
	NaCl	***	*	**	**	***	*
	ASA×NaCl	**	**	**	***	**	***

Significant differences are marked with asterisks, as follows: ∗*P<* 0.05, ∗∗*P<* 0.01, and ∗∗∗ *P<* 0.001. Differences with *p >* 0.05 are regarded as non-significant.

#### 3.5.2 Change in ions contents in the root

**Figure 8:**
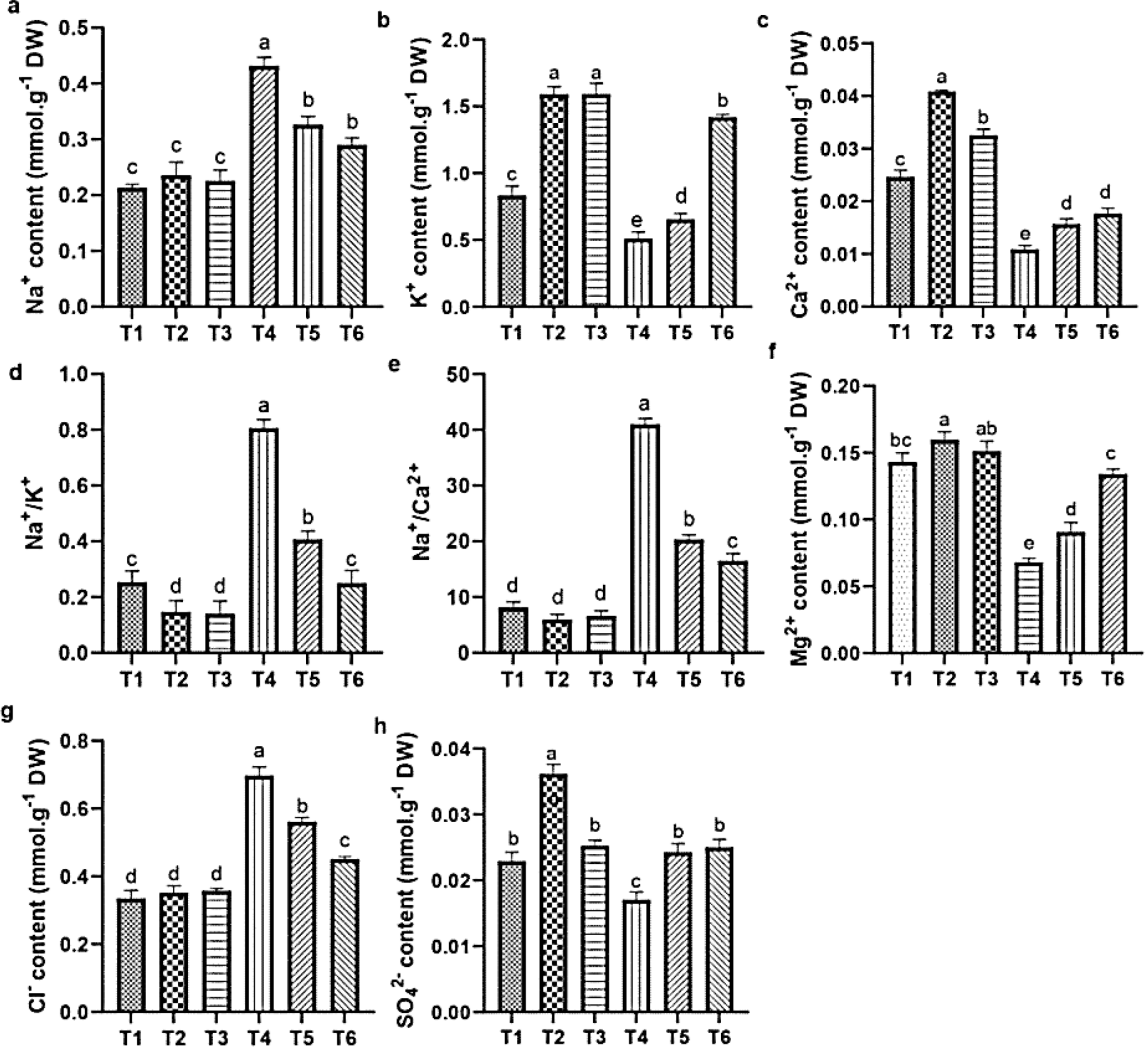
Influence of ASA and salt stress on Na^+^ **(a)**, K^+^ **(b)**, Ca^2+^ **(c)**, Na^+^/K^+^ **(d)**, Na^+^/Ca^2+^ **(e)**, Mg^2+^ **(f)**, Cl^-^ **(g)**, and SO_4_^2-^ **(h)** in quinoa root. Bars represent the mean ± S.E. (n=6); significant variations (*p<* 0.05) are indicated by different letters (a–d).

Salt stress negatively affected the cation and anion contents in quinoa roots. Specifically, salt stress increased the level of Na^+^ and Cl^-^ and Na^+^/K^+^ and Na^+^/Ca²^+^ ratios (**Figures 8d, e, g**), while it reduced the K^+^, Ca²^+^, Mg²^+^, and SO_4_²^−^ contents, compared with CK. In contrast, the ASA treatments alleviated these adverse effects. Individually, the ASA treatments significantly increased the K^+^, Ca²^+^, and SO_4_²^−^ levels, while they reduced Na^+^ in roots compared with CK (**Figures 8a–c**). The ASA treatments alone increased K^+^ by 91.25% and 92.43%, respectively, compared with CK. Under salt stress, the ASA treatments significantly increased Mg²^+^ by 94.98% and 33.90%, SO_4_²^−^ by 35.45% and 29.89%, while they decreased Na^+^ by 31.09% and 37.67%, and Cl^-^ by 20.45% and 37.87%, respectively, relative to salt treatment alone (**Figures 8f, h**). The effects of ASA, NaCl, and their interaction significantly influenced the Na^+^, K^+^, Ca²^+^, Mg²^+^, and Cl^-^ levels in quinoa; however, ASA alone did not affect Na^+^ levels (**Table 2**).

### 3.6 The effect of ASA on antioxidant enzymes activities under salt stress

**Figure 9:**
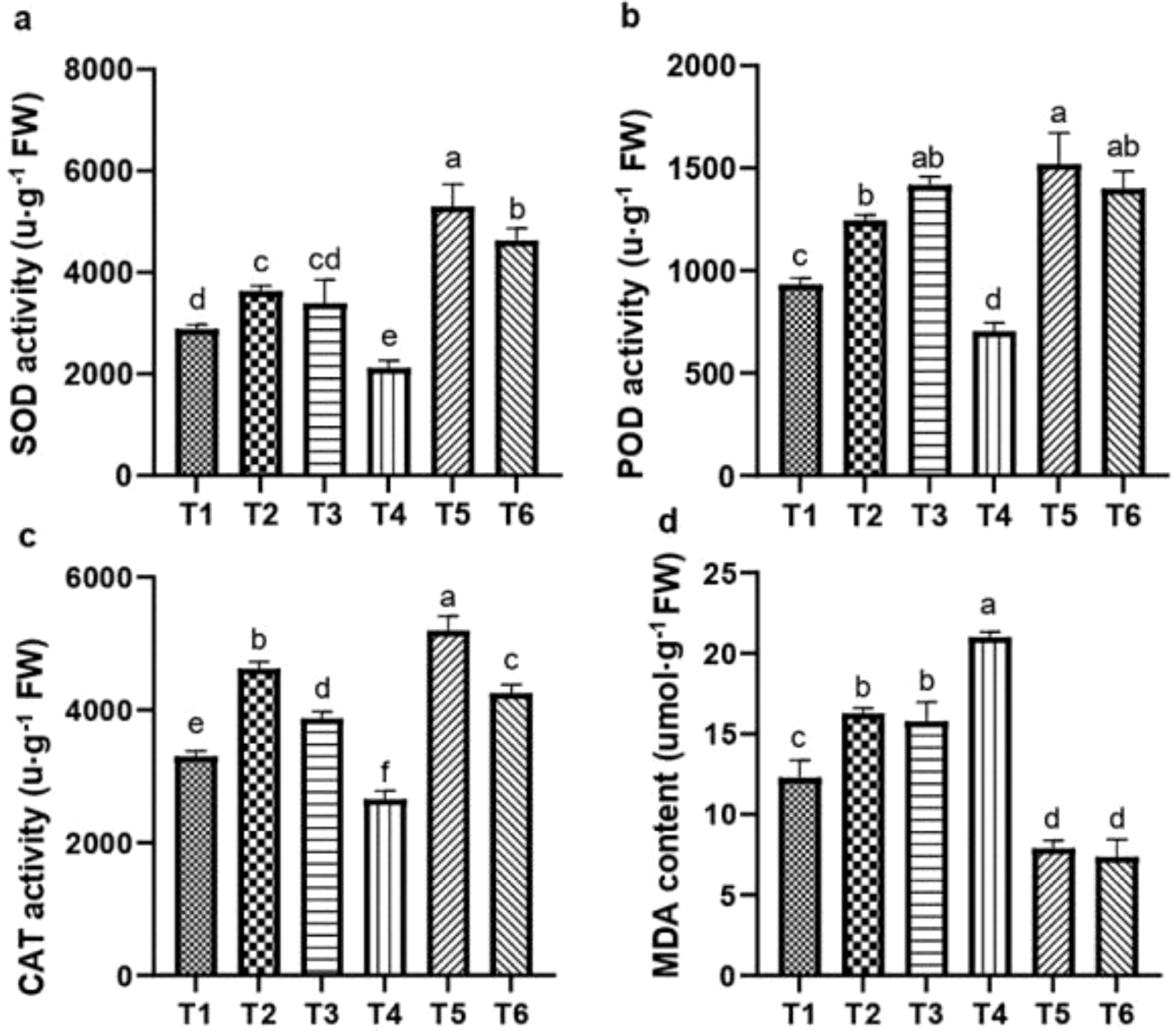
Influence of ASA and salt stress on SOD **(a)**, POD **(b)**, CAT **(c)**, and MDA **(d)** levels in quinoa. Bars represent the mean ± S.E. (n=6); letters (a–d) show significant differences (*P<* 0.05).

Salt stress significantly reduced the activities of SOD, POD, and CAT by 21.57%, 23.89%, and 17.69%, respectively, and increased the MDA content by 56.90%, relative to CK. However, the effects of salt stress were mitigated by the ASA treatments. Both ASA treatments alone significantly enhanced the SOD activity by 19.27% and 28.25%, POD activity by 31.34% and 47.78%, and CAT activity by 41.56% and 17.21%, respectively, compared to the CK (**Figures 9a–c**). Under salt stress, the ASA treatments significantly increased the SOD activity by 99.43% and 94.88%, POD activity by 98.90%, 95.21%, and CAT activity by 78.55%, 52.86% while they reduced MDA contents by 62.35% and 64.67%, respectively, compared with the salt stress alone (**Figure 9d**).

### 3.7 Principal component analysis

**Figure 10:**
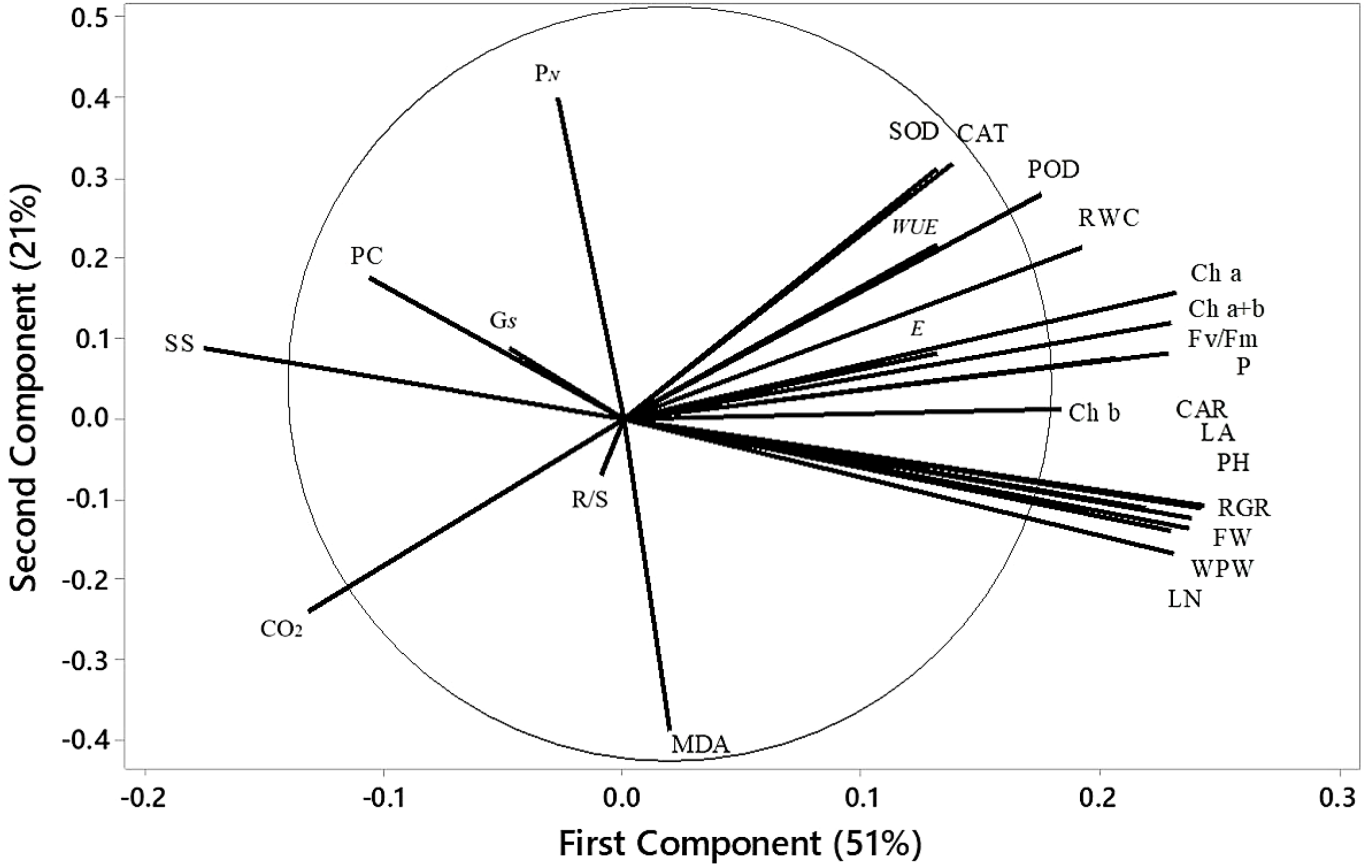
Loading plot of PCA depicting the physiological indices of quinoa under various treatments. WPW, whole plant weight; LN, leaf number; PH, plant height; FW, fresh weight of shoots; RGR, relative growth rate; LA, leaf area; R/S, root-to-shoot ratio; Ch a, chlorophyll a; Ch b, chlorophyll b; Ch a+b, total chlorophyll content; CAR, carotenoids; RWC, relative water content; *P_N_*, net photosynthesis rate; SS, soluble sugar; *E*, transpiration rate; PC, proline content; G*s*, stomatal conductance.

**Figure 11:**
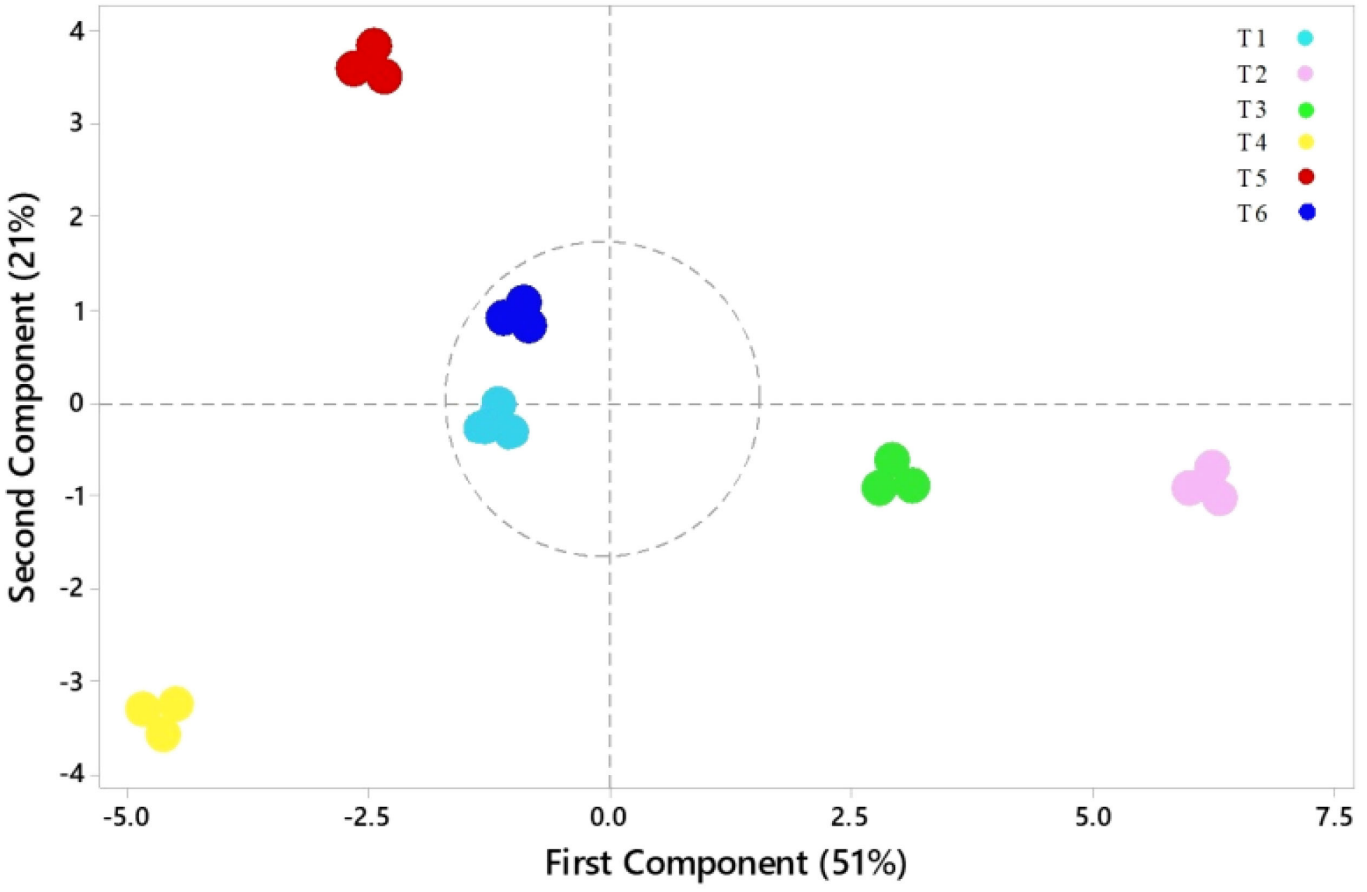
Scores plot from the PCA of the physiological indices of quinoa seedlings under different treatments. T_1_ = control; T_2_ = low ASA (0.1 μM); T_3_ = high ASA (0.5 μM); T_4_ = salt stress (200 mM); T_5_ = low ASA + salt (0.1 μM + 200 mM); T_6_ = high ASA + salt (0.5 μM + 200 mM).

Variations among all parameters were analyzed by principal component analysis (PCA), which is shown in **Figure 10**. The first principal component (PC1) contributed 51% of the total variance, and the second principal component (PC2) contributed 21%, capturing 72% of the total variation in the dataset. These two principal components thus effectively summarize the majority of the information from the original 18 indicators. The PCA transformed the initial set of indicators into two distinct, uncorrelated comprehensive components. PC2 effectively separated parameters such as CO_2_, R/S, and MDA, which exhibited a strong negative correlation with parameters such as antioxidants, chlorophyll content, and specific biomass indices, including *WUE*. In contrast, PC1 revealed positive correlations among most biomass-related parameters, such as LN, FW, and RGR. The score plot (**Figure 11**) showed that treatment T_5_ had the highest positive score, while T_4_ exhibited the most negative scores on PC1 and PC2. The control treatment showed PC1 scores close to zero, whereas treatments T_2_ and T_3_ exhibited positive scores on PC1, indicating a resilient response of ASA to salt stress. Treatment T_6_, which involved high salt and ASA conditions, had a positive score on PC2 compared with the control. Overall, T_5_ displayed the optimal performance, followed by T_6_, suggesting that ASA may help alleviate the impact of salt stress while enhancing antioxidant machinery and physiological processes.

## 4 Discussion

Salt stress inhibits seed germination and growth by reducing water and osmotic potential, adversely affecting the embryo and sprout (Otie, 2022; Begum et al., 2022). It also impairs seed germination and development in essential crops (Otie, 2022; Chakma et al., 2019; Chauhan, 2022). The results of this study indicate that the observed decreases in GP, GE, GRI, SP, and SVI can be attributed to osmotic stress and ion toxicity, disrupting water absorption and altering the nutrient balance, thus reducing seedling development and germination, as noted previously by Mittal et al. (2018). These findings align with the conclusion of Chen HAKM. et al. (2021), who highlighted the harmful effect of salt stress on seed germination and seedling development. To counteract these effects, ASA was applied at 0.1 µM and 0.5 µM concentrations, selected based on earlier research showing they effectively mitigated salt-induce stress. Notably, the lower ASA dose markedly improved SVI and GRI (**Figures 6c, e**), suggesting its role in enhancing early seedling vigor by regulating osmotic balance and reducing ROS-induced damage. Previous research on tomatoes (*Solanum lycopersicum*) and mustard (*Brassica rapa*) under salinity stress has shown that both exogenous and endogenous ASA application enhances germination parameters (Mittal et al., 2018; Chen et al., 2023). In these studies, the improvement in germination is attributable to the role of ASA as an antioxidant, mitigating oxidative damage and enhancing osmotic balance. The results of this study further corroborate the findings of Zhang G. et al. (2019) and Chan et al. (2013), which showed that exogenous ASA promotes cell growth, root development, and seedling growth while mitigating the detrimental impacts of salinity on seed germination of wheat and okra (*Abelmoschus esculentus* L.). However, the concentration and duration of ASA application are critical, as optimal levels improve germination, while improper dosages may induce toxicity (Sarkar et al., 2023). The experimental analysis revealed that a lower ASA dose is particularly effective in improving quinoa seed germination and seedling development (**Tables 3**, **4**), probably by mitigating oxidative stress and enhancing antioxidant defenses.

**Table 3:** Effects of different ASA and salt stress treatments on quinoa seed radicle length, measured at six-hour intervals over five days.

Time (hours)	T_1_ Control	T_2_ ASA 0.1	T_3_ ASA 0.5	T_4_ NaCl 200	T_5_ ASA 0.1+Salt 200	T_6_ ASA 0.5+Salt 200
6h	0.04 ± 0.0516 ab	0.09 ± 0.0738 a	0.06 ± 0.0699 ab	0.01 ± 0.0316 b	0.03 ± 0.0483 ab	0.02 ± 0.0422 ab
12	0.2 ± 0.0667 b	0.31 ± 0.1269 a	0.21 ± 0.1265 b	0.14± 0.0699 b	0.22 ± 0.0667 b	0.22 ± 0.0675 b
18	0.4 ± 0.0516 b	0.65 ± 0.1418 a	0.44± 0.0738 b	0.26 ± 0.0527 c	0.4 ± 0.0527 b	0.43± 0.0632 b
24	0.64 ± 0.1101 b	0.83 ± 0.1494 a	0.61 ± 0.0919 b	0.35 ± 0.0483 c	0.55 ± 0.0738 b	0.58 ± 0.0789 b
30	0.91 ± 0.137 b	1.33 ± 0.1494 a	0.78 ± 0.0738 c	0.47 ± 0.0422 d	0.71 ± 0.0675 c	0.72 ± 0.0816 c
36	1.21 ± 0.137 b	1.77 ± 0.1494 a	0.91 ± 0.0738 c	0.62 ± 0.0422 d	0.83 ± 0.0675 c	0.8 ± 0.0816 c
42	1.59 ± 0.1969 b	2.16 ± 0.1173 a	1.31 ± 0.1370 c	0.77 ± 0.0948 e	1.07 ± 0.0948 d	1.1 ± 0.1333 d
48	1.68 ± 0.1833 b	2.27 ± 0.1251 a	1.41 ± 0.1370 c	0.88 ± 0.1135 e	1.18 ± 0.1032 d	1.2 ± 0.1333 d
54	1.94 ± 0.1173 b	2.39 ± 0.1100 a	1.5 ± 0.1247 c	0.93 ± 0.1159 e	1.23 ± 0.0674 d	1.25 ± 0.1178 d
60	2.13 ± 0.1337 b	2.56 ± 0.1429 a	1.52 ± 0.1135 c	0.93 ± 0.0159 e	1.23 ± 0.0674 d	1.25 ± 0.1178 d
66	2.46 ± 0.1264 b	2.92 ± 0.1581 a	1.57 ± 0.0948 c	0.98± 0.0918 e	1.24 ± 0.0514 d	1.27 ± 0.0948 d
72	2.55 ± 0.0971b	3.11 ± 0.1100 a	1.62 ± 0.7800 c	1.07 ± 0.0674 e	1.33 ± 0.0674 d	1.32 ± 0.0788 d
78	2.76 ± 0.1349 b	3.64 ± 0.1349 a	1.66 ± 0.0516 c	1.07 ± 0.0674 e	1.34 ± 0.0516 d	1.35 ± 0.0707 d
84	2.99 ± 0.0737 b	4.32 ± 0.1316 a	1.7 ± 0.0816 c	1.11 ± 0.0875 e	1.37 ± 0.0674 d	1.36 ± 0.0699 d
90	3.21 ± 0.0875 b	4.47 ± 0.1702 a	1.7 ± 0.0816 c	1.11 ± 0.0875 e	1.38 ± 0.0632 d	1.38 ± 0.0918 d
96	3.43 ± 0.1059 b	4.83 ± 0.1567 a	1.7 ± 0.0816 c	1.11 ± 0.0875 e	1.38 ± 0.0632 d	1.39 ± 0.0875 d
102	4.33 ± 0.1059 b	5.93 ± 0.1636 a	1.72 ± 0.0788 c	1.12 ± 0.0788 e	1.44 ± 0.0151 d	1.42 ± 0.0918 d
108	5.17 ± 0.0674 b	7.08 ± 0.1475 a	1.78 ± 0.0632 c	1.13 ± 0.0674 e	1.46 ± 0.0151 d	1.45 ± 0.0701 d
114	6.08 ± 0.0788 b	8.17 ± 0.0948 a	1.79 ± 0.0737 c	1.13 ± 0.0674 e	1.46 ± 0.0151 d	1.47 ± 0.0674 d
120	6.27 ± 0.0483 b	8.39 ± 0.1286 a	1.89 ± 0.0737 c	1.17 ± 0.0674 e	1.51 ± 0.0737 d	1.48 ± 0.0632 d

These data are expressed as the mean ± S.E. with three replicates per treatment. Tukey’s-b test was applied to assess significance, where distinct letters (a-f) denote statistically significant variances at *P<* 0.05.

**Table 4:** Effects of different ASA and salt stress treatments on quinoa seed plumule length, measured at six-hour intervals over five days.

Time (hours)	T_1_ Control	T_2_ ASA 0.1	T_3_ ASA 0.5	T_4_ NaCl 200	T_5_ ASA 0.1+Salt 200	T_6_ ASA 0.5+Salt 200
6h	0	0	0	0	0	0
12	0	0	0	0	0	0
18	0	0	0	0	0	0
24	0	0.20 ± 0.0632 a	0	0	0	0
30	0.08 ± 0.1135 bc	0.37 ± 0.0675 a	0.10 ± 0.1155 b	0	0	0
36	0.40 ± 0.1054 b	0.66 ± 0.1075 a	0.47 ± 0.0949 b	0	0	0
42	0.70 ± 0.1054 b	1.11 ± 0.1247 a	0.75 ± 0.0850 b	0	0.07 ± 0.1160 c	0.03 ± 0.0675 c
48	0.80 ± 0.1054 b	1.22 ± 0.1033 a	0.86 ± 0.1075 b	0.07 ± 0.1160 d	0.22 ± 0.0919 c	0.15 ± 0.1179 cd
54	0.99 ± 0.0876 c	1.36 ± 0.0699 a	1.24 ± 0.0699 b	0.30 ± 0.0666 d	0.37 ± 0.0674 d	0.36 ± 0.0966 d
60	1.19 ± 0.0738 c	1.50 ± 0.0816 a	1.34 ± 0.0699 b	0.40 ± 0.0666 e	0.73 ± 0.0942 d	0.68 ± 0.1135 d
66	1.37 ± 0.0823 c	1.91 ± 0.0994 a	1.63 ± 0.0823 b	0.50 ± 0.0666 e	0.99 ± 0.1286 d	0.73 ± 0.0674 d
72	1.47 ± 0.0823 c	2.06 ± 0.0843 a	1.73 ± 0.0823 b	0.59 ± 0.0567 f	1.11 ± 0.1100 d	0.81 ± 0.0737 e
78	169 ± 0.1197 c	2.20 ± 0.1054 a	1.90 ± 0.0666 b	0.68 ± 0.0632 f	1.15 ± 0.0707 d	0.85 ± 0.0527 e
84	1.89 ± 0.0875 c	2.36 ± 0.0843 a	2.13 ± 0.0848 b	0.79± ± 0.0737 f	1.22 ± 0.0632 d	0.95 ± 0.0849 e
90	1.99 ± 0.0875 c	2.46 ± 0.0843 a	2.20 ± 0.0666 b	0.83 ± 0.0674 f	1.25 ± 0.0707 d	0.95 ± 0.0849 e
96	2.08 ± 0.0788 c	2.61 ± 0.0994 a	2.26 ± 0.0843 b	0.89 ± 0.0994 f	1.28 ± 0.0632 d	1.01 ± 0.0875 e
102	2.14 ± 0.0516 c	2.71 ± 0.0994 a	2.26 ± 0.0843 b	0.91 ± 0.0737 f	1.31 ± 0.0567 d	1.04 ± 0.0843 e
108	2.23 ± 0.0483 c	2.81 ± 0.09944 a	2.35 ± 0.0707 b	0.94 ± 0.0788 f	1.36 ± 0.0516 d	1.08 ± 0.1032 e
114	2.31 ± 0.0567 b	2.91 ± 0.09944 a	2.37 ± 0.0674 b	0.92 ± 0.0699 e	1.38 ± 0.0632 c	1.12 ± 0.0918 d
120	2.39 ± 0.0567 b	3.11 ± 0.09944 a	2.45 ± 0.0707 b	0.96 ± 0.0516 e	1.41 ± 0.0567 c	1.14 ± 0.0699 d

These data are expressed as the mean ± S.E. for three replicates per treatment. Statistical significance was determined using the Tukey’s-b test, with distinct letters (a-f) denoting statistically significant variations at *P<* 0.05.

Salt stress hampers plant development and growth by disrupting osmotic balance and inducing ion toxicity (Sardar, 2023; Gupta and Huang, 2014). A plethora of studies have confirmed that salt stress significantly hampers plant growth, productivity, and yield by inducing Na^+^ toxicity (Biswas et al., 2023; Sardar, 2023). It also disrupts photosynthesis by causing stomatal closure, which reduces intercellular CO_2_ concentrations, and interferes with the transport of essential ions (Najafi Zilaie et al., 2023). Similarly, the results of this study indicate that salt stress significantly reduced RGR, FW, DW, R/S, LN, PH, and LA. These effects could be attributed to disruptions in osmotic balance, impaired water uptake, and ion toxicity, all hindering quinoa growth. The reduction in FW and DW (**Figures 2c, d**), underscores the inhibitory impact of salt stress on biomass accumulation, primarily due to impaired water uptake and disrupted nutrient homeostasis as can be seen in RWC results (**Figure 2b**). The reduction is closely associated with osmotic stress, which disrupts metabolic processes, impacting cell growth, division, and the efficiency of photosynthesis (Chen HAKM. et al., 2021). Understanding the mechanisms behind these physiological disruptions is key for developing effective agronomic strategies to enhance salt tolerance. However, several studies such as El-Afry et al. (2018), and Azeem (2023) have demonstrated that applying ASA improves plant growth, photosynthesis, stomatal conductance, oxidative defense, and the levels of photosynthetic pigments. The improvement in RGR and PH (**Figures 2a, e**) in our study suggests that ASA significantly supports cell expansion and elongation under stress conditions. In addition, the increase in LN and large LA in ASA-treated plants (**Figures 2f, g**) indicate improved photosynthetic efficiency and carbon assimilation. Interestingly, the expectation that growth parameters such as FW, DW per plant, and LN of ASA-treated plants would be lower than CK plants under salt stress, was not supported as shown in **Figure 2**. This could be due to the antioxidant properties of ASA, which may have alleviated the negative impacts of salt stress. Additionally, the applied salt concentration (200 mM) may not have been high enough to induce severe toxicity in the quinoa cultivar Longli No.1, which is known for its tolerance to salinity levels between 200–400 mM (Yang et al., 2017). Therefore, the observed slower growth in salt treated plants compared to CK may still reflect a mild salt stress effect, particularly in the absence of ASA. In contrast, ASA treated plants likely benefited from enhanced antioxidant defense, improved nutrient uptake, and photosynthetic performance, resulting in better biomass accumulation even under 200 mM salt. These findings suggest that the combination of a salt-tolerant and ASA application may partially overcome growth limitations typically associated with salt stress. Moreover, the observed interaction between salt stress and ASA treatment highlights the complex and adaptive physiological responses of quinoa under high-salinity conditions. The findings are consistent with previous reports and further support the hypothesis that ASA enhances quinoa growth by facilitating photosynthesis, ion homeostasis, biochemical adjustments and growth under salt stress (El-Sayed and El-Sayed, 2013).

Leaf gas exchange, photosynthetic pigments, and chlorophyll *a* fluorescence are key indicators of plant health and stress tolerance (Nouman and Aziz, 2022; Bernier Brillon et al., 2023). These parameters are often reduced in saline environments because of disrupted chlorophyll biosynthesis, impaired metabolic processes, and damage to the photosynthetic apparatus caused by ion toxicity and osmotic stress (Swapna, 2003; Askari, 2023). In the current study, salt stress significantly reduced chlorophyll contents, which could be attributed to disrupted pigment biosynthesis and instability. Similarly, in accordance with our results, salt stress negatively impacts the chlorophyll content in tomatoes, carotenoids in milk thistle (*Silybum marianum*), stomatal conductance in common bean (*Phaseolus vulgaris*), and the Fv/Fm ratio in sweet peppers, all reflecting significant impairments associated with salt stress (El-Sayed and El-Sayed, 2013; Ekmekçi and Karaman, 2012; Gaafar, 2020; El-Beltagi, 2022). Recent studies on broccoli (*Brassica oleracea*), sweet pepper, and rice have shown that applying ASA enhances photosynthetic efficiency by improving the performance of PSII, pigment contents, electron transport, and chloroplast membrane protection (MacDonald et al., 2022; El-Beltagi, 2022; Chen HAKM. et al., 2021). El-Sayed and El-Sayed (2013) reported that exogenous ASA stimulates the enzymes involved in chlorophyll and carotenoid biosynthesis, leading to higher photosynthetic pigment levels under salt stress. Similarly, our results support the aforementioned findings, as higher chlorophyll levels were maintained in ASA-treated plants under salt stress (**Figures 4a–c**). Furthermore, a study on chicory (*Cichorium intybus* L.) found that ASA increased *P_N_* and *g_s_* (Sadia et al., 2023). In our results, P*_N_* and *g_s_* also improved, indicating enhanced stomatal regulation and CO_2_ assimilation. Likewise, the increase in *WUE* suggests that ASA treated plants maintained better water use efficiency under stress conditions. Chen et al. (2023) demonstrated that exogenous ASA mitigates the inhibition of Fv/Fm, PSII quantum yield, and NPQ values under salt stress. The same results were found in our study, especially the improvement in NPQ, (**Figure 4f**), suggesting that ASA enhanced energy dissipation mechanisms, which is crucial for preventing photodamage and dissipating excess energy under salinity stress. Also, qP and ETR (**Figures 4e, g**) showed that ASA enhanced electron transport efficiency, supporting sustained photosynthetic activity under stress conditions. Furthermore, Henschel et al. (2023) found that applying ASA increased the levels of soluble proteins, sugars, and proline, as well as the transpiration rate, all of which could contribute to improved photosynthetic efficiency. Soluble sugar content was markedly increased in our study, indicating enhanced osmotic adjustment and energy reserves under salinity conditions. Our results also indicate that ASA induced sugar accumulation varies with treatment intensity, suggesting a threshold effect in osmotic regulation. Similarly, proline accumulation was significantly higher in ASA combined treatments, suggesting its role in osmotic balance and ROS scavenging (**Figure 6b**). These effects are attributable to the higher priming ability of ASA to enhance antioxidant defenses, maintain cellular function, and protect the photosynthetic apparatus from stress-induced damage (Koleva-Valkova and Harizanova, 2019). The findings support these conclusions, likely because of the antioxidant properties of ASA. The results align with previous studies on wheat, sorghum (*Sorghum bicolor*), chicory, and marigold (*Tagetes* spp) (Mohammed Ibrahim Elsiddig et al., 2022; Ishaq et al., 2021; Sadia et al., 2023; Azizi et al., 2021). However, the results of this study contradict those of Zonouri et al. (2014), who found no notable impact of exogenous ASA on the pigments involved in photosynthesis under high salinity conditions.

Exogenous ASA has been found to improve physiological functions and boost non-enzymatic and enzymatic antioxidant pathways under saline stress (Alamri et al., 2018; Ekmekçi et al., 2021). In this study, the ASA treatment significantly enhanced antioxidant enzymes activities (POD, CAT, and SOD) and reduced MDA levels under salt stress. The adverse impact of salt-induced stress on quinoa could be attributable to an increase in ROS production that causes oxidative damage and lipid peroxidation (Hasaneen et al., 2010; Shah et al., 2020). However, ASA mitigated these adverse effects by scavenging ROS, regulating the redox balance, and activating antioxidant enzymes. Foliar application of ASA at 5 mM significantly mitigated the negative effects of salt stress and increased enzymatic antioxidants i.e., SOD (22.3%), POD (34.1%) and CAT (39%) in pea (*Pisum sativum* L.) plants (Kanwal et al., 2024). The same results were observed in our findings, where ASA treatments, especially ASA combined with salt stress, induced the strongest antioxidant response in quinoa (**Figures 9a, c**). Additionally, a significant declined in MDA content at low ASA concentrations underscores its role in reducing lipid peroxidation under salt stress. Similarly, ASA application effectively protected okra seedlings by enhancing growth, photosynthetic pigments, and antioxidant activities while reducing the hydrogen peroxide and lipid peroxidation under salt stress (Zhang C. et al., 2019). In barley (*Hordeum vulgare*), ASA enhanced antioxidant production and reduced lipid peroxidation due to its capacity to neutralize ROS and protect cellular components from oxidative damage (Talaat et al., 2023). Furthermore, Naz et al. (2013) reported that ASA reduces oxidative stress by synergizing with other antioxidants to regulate key enzymes, thereby managing oxidative damage and maintaining cellular balance. These findings aligned with the results from wheat (El-Hawary et al., 2023), rice (Che Radziah et al., 2016), sugarcane (El-Hawary et al., 2023), and sorghum (Mohammed Ibrahim Elsiddig et al., 2022), where exogenous ASA treatments significantly enhanced SOD, POD, and CAT activities while decreasing MDA levels. Although ASA levels were not quantified in this study, it is acknowledged that measuring endogenous ASA content could have provided further insight into its direct role as a non-enzymatic oxidant in ROS detoxification. In addition to understanding its distribution within plant tissues, such data would allow a more precise evaluation of its active participation in oxidative stress regulation. Future studies should consider this aspect to strengthen the mechanistic understanding of ASA role under salt stress.

**Figure 12:**
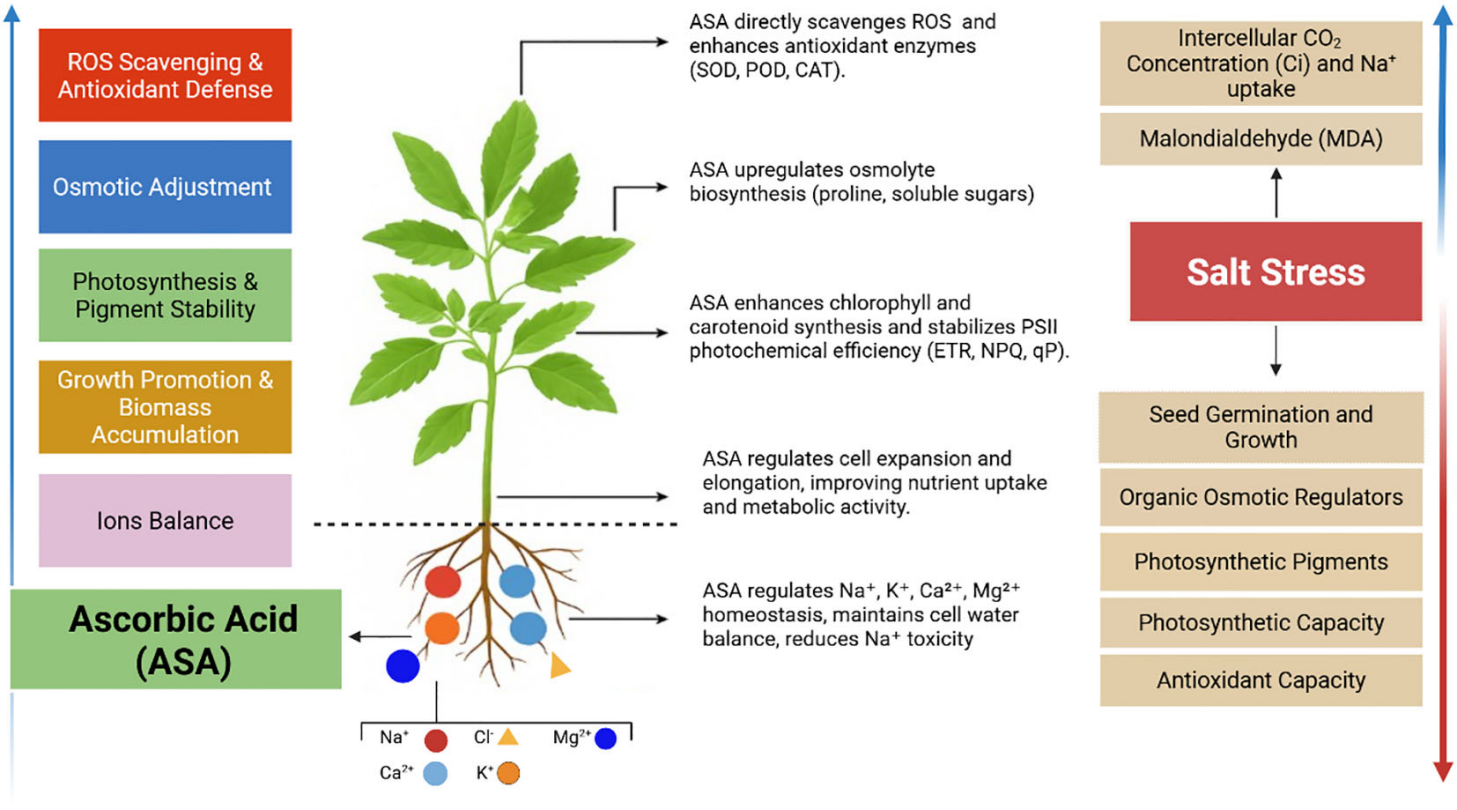
Changes in physiological and biochemical processes under ASA in quinoa in tolerating salt stress tolerance by enhancing and suppressing paraments found in our results.

Excess salt ions hinder the uptake of essential nutrients and induce osmotic stress in plants (Alamri et al., 2018; Zhang C. et al., 2019). The results showed reduced levels of essential ions and elevated Na^+^ levels in both leaves and roots, inducing a nutrient imbalance, as indicated by higher Na^+^/K^+^ and Na^+^/Ca^2+^ ratios. This imbalance may be due to the competitive uptake of Na^+^ over these essential ions under salt stress. Consistent with Riaz (2020), high Na^+^ levels disrupt the availability of K^+^, Ca^2+^, Mg^2+^, and SO_4_^2-^ for plants. However, ASA treatments alleviated these adverse effects by reducing Na^+^ accumulation and restoring the balance of essential ions especially K^+^ and Ca²^+^, in both leaves and roots as shown in our results (**Figures 7b, c**, **8b, c**). This restoration of ion homeostasis an important finding, as it highlights the potential role of ASA enhancing ion selectivity and transport under stressful conditions such as high salinity levels. Especially, notable is ASA’s ability to enhance K^+^ uptake, as observed in alfalfa (*Medicago sativa* L.) (Chen Z. et al., 2021). Similarly, ASA protects wheat plants from salt stress by improving K^+^ uptake (Zhang G. et al., 2019). Moreover, ASA’s ability restore of Ca²^+^ levels is crucial, as Ca²^+^ supports cell wall integrity and stress signaling under salinity stress (Zhang G. et al., 2019). Similar benefits have been observed in rice (Che Radziah et al., 2016), rapeseed (*Brassica campestris* cv) (Rainhan et al., 2023), and peanut (*Arachis hypogea* L.) (Oliveira et al., 2022), where ASA improves ion transport and selectivity while alleviating salt-induced ion imbalances via its antioxidant properties. In our results, ASA demonstrated a dual role, as it not only reduced Na^+^ toxicity, but also boosted the uptake and retention of essential ions, as mentioned above. This dual mechanism combined ASA’s antioxidant properties offers an integrated strategy to mitigate salt-induced inhibition in quinoa. Thus, the alleviation of quinoa growth inhibition under salt-induced stress can be linked to improved ion balance facilitated by ASA, particularly at a concentration of 0.1 μM. The applied ASA concentration and mode of exogenous application influence plant growth, stress tolerance, and overall resilience, as illustrated in **Figure 12**. However, this study focused on a single salt concentration (200 mM), and future research should explore a broader range, including 300, 400, and 500 mM, to test ASA in quinoa and other crops. Additionally, the effects of ASA throughout the full quinoa life cycle, from germination to harvest, should be examined, since this study only focused on the seedling stage. Moreover, this study considered a single growth season, so long-term studies across multiple seasons are needed to determine the sustainability and effectiveness of ASA under various soil conditions. Finally, although ASA improved ion homeostasis and osmotic adjustment, its effects on other critical growth parameters, such as root development and yield formation, require further investigation.

## 5 Conclusions

In the current study, salt stress inhibited seed germination, growth traits, photosynthetic pigments, chlorophyll *a* fluorescence, ion balance, and antioxidant activities in quinoa. However, ASA treatments at (0.1, 0.5 µM) significantly mitigated these adverse effects, with 0.1 µM exhibiting the most pronounced improvements. At this optimal concentration, ASA enhanced seed germination indices (SIV and GRI) by regulating osmotic balance and reducing ROS-induced damage. It also maintained photosynthetic pigments and improved PH, LN, and LA, suggesting its important role in promoting cell expansion, elongation, and photosynthetic efficiency. Moreover, ASA enhanced NPQ, ETR, and qP, indicating its role in stabilizing photosynthetic machinery under salt stress. Additionally, ASA-mediated upregulation of proline and soluble sugar biosynthesis contributed to osmotic adjustment and stress tolerance. Furthermore, ASA facilitated ion homeostasis and enhanced photosynthetic efficiency, further reinforcing its potential as a stress-mitigating agent. Statistically, the results indicated that the 0.1 µM ASA mitigated the adverse effects of salinity-induced stress, thus improving growth parameters, photosynthetic efficiency, chlorophyll content, and ion balance. Overall, this study offers important insights into the potential application of ASA as a stress-mitigating agent for quinoa cultivation and other crops subjected to salt stress. The findings suggest that ASA plays a critical role in enhancing germination, growth, and stress tolerance during the sensitive stages of quinoa development under salt stress. These results encourage further investigation into the role of ASA during the sensitive stages of crop growth and yield performance under varying salinity and climatic conditions. Based on these findings, it is recommended to incorporate ASA as a biostimulant in agricultural practices for quinoa exposed to saline stress. Future research should focus on long-term field studies to assess the practical application of ASA for quinoa under different saline-alkaline conditions. In addition, investigations into the molecular mechanisms underlying its protective effects will provide deeper insights into its mechanism of action.

## Data Availability

The original contributions presented in the study are included in the article/supplementary material. Further inquiries can be directed to the corresponding authors.
