# Correction: Priming effect of ascorbic acid on the growth and biomass of quinoa under saline conditions

**DOI:** 10.3389/fpls.2025.1644150

**Published:** 2025-07-02

**Authors:** Frontiers Production Office

**Affiliations:** Frontiers Media SA, Lausanne, Switzerland

**Keywords:** ascorbic acid, *Chenopodium quinoa*, photosynthetic efficiency, antioxidant response, ion homeostasis, salt stress tolerance

In the published article, there was an error. A grammar mistake was made, the word “improve” should be replaced with “improvement in”.

A correction has been made to **Abstract**, paragraph 1, page 1. This sentence previously stated:

“Furthermore, ASA enhanced gas exchange, non-photochemical quenching (NPQ), and antioxidants enzymes activities, suggesting improve energy dissipation and potential support for oxidative stress tolerance”.

The corrected sentence appears below:

“Furthermore, ASA enhanced gas exchange, non-photochemical quenching (NPQ), and antioxidants enzyme activities, suggesting improvement in energy dissipation and potential support for oxidative stress tolerance”.

The original version of this article has been updated.

